# Quantifying the regulatory role of individual transcription factors in *Escherichia coli*

**DOI:** 10.1016/j.celrep.2021.109952

**Published:** 2021-11-09

**Authors:** Sunil Guharajan, Shivani Chhabra, Vinuselvi Parisutham, Robert C. Brewster

**Affiliations:** 1 Department of Systems Biology, University of Massachusetts Chan Medical School, Worcester, MA 01605, USA; 2 Department of Pharmacological Sciences, Icahn School of Medicine at Mount Sinai, New York, NY 10029, USA; 3 Department of Microbiology and Physiological Systems, University of Massachusetts Chan Medical School, Worcester, MA 01605, USA; 4 Lead contact

## Abstract

Gene regulation often results from the action of multiple transcription factors (TFs) acting at a promoter, obscuring the individual regulatory effect of each TF on RNA polymerase (RNAP). Here we measure the fundamental regulatory interactions of TFs in *E. coli* by designing synthetic target genes that isolate individual TFs’ regulatory effects. Using a thermodynamic model, each TF’s regulatory interactions are decoupled from TF occupancy and interpreted as acting through (de)stabilization of RNAP and (de)acceleration of transcription initiation. We find that the contribution of each mechanism depends on TF identity and binding location; regulation immediately downstream of the promoter is insensitive to TF identity, but the same TFs regulate by distinct mechanisms upstream of the promoter. These two mechanisms are uncoupled and can act coherently, to reinforce the observed regulatory role (activation/repression), or incoherently, wherein the TF regulates two distinct steps with opposing effects.

## In brief

Guharajan et al. investigate the isolated regulation of 6 *E. coli* transcription factors. The diverse regulatory outcomes are well described by a model wherein TFs act on two different steps of transcription. The degree to which each step is regulated depends on TF identity and binding location.

## Graphical Abstract

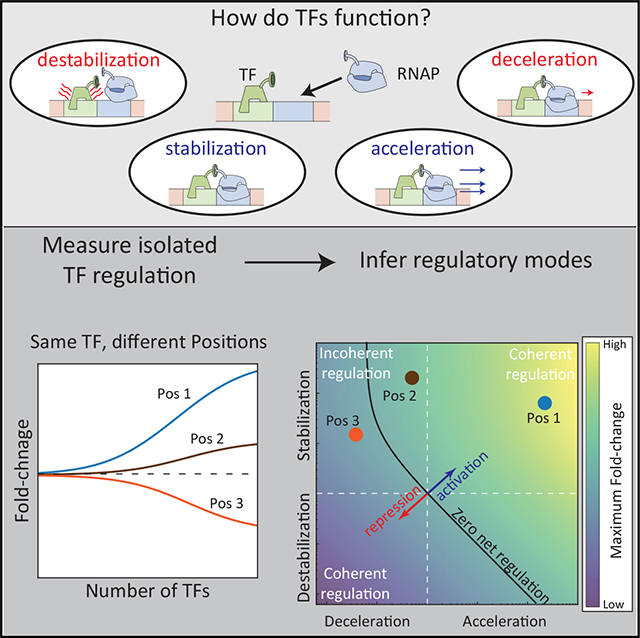

## INTRODUCTION

Transcriptional regulation of gene expression is one of the major mechanisms by which cells respond to cues and stimuli. Transcription factors (TFs) perform this regulation through binding to the DNA around the promoter to alter the rate of transcription from individual genes (Jacob and Monod, 1961; Ptashne and Gann, 2002). The regulatory DNA of each gene is distinct and can involve several to dozens of TF binding sites arranged in specific architectures to achieve the desired expression level. However, predicting the level of gene expression based on the regulatory architecture of a gene remains a central challenge in the field (Guido et al., 2006; Sprinzak and Elowitz, 2005; Atkinson et al., 2003; Nielsen et al., 2016; Ali et al., 2020).

The genomics era has enabled multiple techniques capable of determining where a TF will bind and with what specificity (Stormo, 2000; Messeguer et al., 2002; Wasserman and Sandelin, 2004; Weirauch et al., 2014). Although this information is crucial for building occupancy-based models of gene regulation, there is still another critical component that is missing; the quantitative regulatory role of a TF, when bound, is often unclear. Historically, measurements of TF function are the result of knocking out the endogenous TF and observing how gene expression changes as a result. This serves a purpose in predicting the specific role of that TF on a given gene but offers less predictive power when examining regulation of other genes by that same TF or to different binding sites. These measurements of gene regulation are often entangled in indirect regulatory effects such as TF-TF interactions (Vidal et al., 2011; Rolland et al., 2014), feedback (Bateman, 1998; Shen-Orr et al., 2002), and physiological (i.e., growth rate) (Klumpp et al., 2009; Schmidt et al., 2016) and off-target competitive effects of decoy binding sites or other genes in the network (Brewster et al., 2014; Lee and Maheshri, 2012). Because of this, a single TF that binds at the same relative location on two different natural promoters can appear to have opposite regulatory roles. The entanglement between indirect and direct regulation likely contributes to this ambiguity and prevents the field from developing a basic intuition of TF regulatory function. The select few TFs that have arisen as “model TFs,” such as LacI (Oehler et al., 1990; Garcia and Phillips, 2011; Hammar et al., 2014; Daber et al., 2011; Vilar and Saiz, 2013), AraC (Lobell and Schleif, 1990; Schleif, 2010; Egan et al., 2000; Egan, 2002), lambda repressor (Ptashne et al., 1980; Shea and Ackers, 1985; Ptashne, 2004), CRP (Gaston et al., 1989; Ushida and Aiba, 1990; Kolb et al., 1993; Kinney et al., 2010; Forcier et al., 2018), and TetR (Ramos et al., 2005; Deng et al., 2013; Stanton et al., 2014; Gardner et al., 2000), have well-studied regulatory function. Indeed, these TFs have been utilized for design of synthetic circuits with an engineered purpose, such as creation of logic gates (Stanton et al., 2014; Anderson et al., 2007), bistable switches (Gardner et al., 2000; Tan et al., 2009), oscillatory networks (Elowitz and Leibler, 2000; Stricker et al., 2008; Atkinson et al., 2003), synthetic enhancers (Amit et al., 2011; Brunwasser-Meirom et al., 2016), and a host of other dynamic outcomes. Further characterizing the regulatory function of TFs beyond this small subset should provide a more complete toolset for broader synthetic design purposes.

Here we study the isolated regulatory function of a set of *E. coli* TFs in a system designed to remove the typical confounding factors of natural genes and quantify the direct regulatory effect of a TF based on factors such as TF concentration, binding affinity, and binding location. Using a collection of strains where the average copy number of most TFs in the cell can be controlled, we measure the level of regulation of an individual TF acting on a synthetic promoter sequence. This promoter is designed to be regulated only by that TF, and it is targeted to a binding site whose location and sequence we control. To interpret these data, we use a thermodynamic model of gene regulation to parameterize TF regulatory function. In principle, the TF could exert its regulatory effect at any one of the distinct kinetic steps of the transcriptional process (Kontur et al., 2008; Revyakin et al., 2006; Young et al., 2002; Henderson et al., 2017; Jensen and Galburt, 2021) or on several of them, and our model coarse-grains TF activity into two distinct modes of regulation. The first regulatory mode, “stabilization,” corresponds to stabilization (or destabilization) of the polymerase at the promoter by the TF and models the TF’s ability to facilitate the emergence of the closed RNA polymerase (RNAP)-DNA complex. Essentially, this reflects a modification of the off-rate (*k*_*off*_*)* of bound RNAP when the TF is co-bound at the promoter, resulting in longer or shorter dwell times of RNAP (β greater than or less than 1) (Roy et al., 1998; Liu et al., 2017). The second mode, “acceleration,” corresponds to a TF’s ability to accelerate (or decelerate) initiation of transcription when the TF and polymerase are bound to the promoter. Canonically, the rate of transcription is dictated by the progress of several intermediate steps during promoter melting (Feklistov, 2013; Feklistov et al., 2017; Boyaci et al., 2019), and the TF’s propensity to accelerate or decelerate transcription can be viewed as acting on the ability of RNAP core subunits to initiate this process (Rhodius and Busby, 2000; Feng et al., 2016; Lee and Maheshri, 2012). Using this model, we infer the quantitative contribution from each of these modes in the data. Importantly, this process allows decoupling of properties that are extrinsic to the TF, such as affinity to the operator binding site, the overall concentration of the TF, or feedback in the network from the core regulatory role of the TF in modulating the steps of the transcription process.

We expect the regulatory parameters of a TF to vary based on the identity of the TF and the binding site location on the regulated promoter. In this study, we investigate the role of TF identity by measuring regulation of 6 TFs (AcrR, AgaR, ArsR, AscG, BetI, and CpxR). These 6 TFs were selected based on their diverse *in vivo* functions, which encompass multidrug resistance (AcrR) (Gu et al., 2008), regulation of metabolic homeostasis (AgaR, AscG, and BetI) (Ishida et al., 2009; Lamark et al., 1996; Ray and Larson, 2004; Leyn et al., 2012), tolerance of heavy metal toxicity (ArsR) (Ren et al., 2017), and coordination of the envelope stress response (CpxR) (DiGiuseppe and Silhavy, 2003; Hews et al., 2019). Furthermore, these TFs encompass 5 distinct TF regulatory families and require different co-factors and allosteric configurations to realize their regulatory function (Salgado et al., 2013). We tested each of these TFs at two common binding locations: directly downstream of the promoter, where repression is commonly observed, and 61 bases upstream of the promoter, a site commonly associated with activation (although databases of regulatory interactions record roughly as many TFs repress at −61 as activate). We find that, despite the diverse nature of the TFs tested (five of the TFs are annotated repressors, and one of them, CpxR, is a known activator), the regulation for all TFs immediately downstream is consistent with a form of repression that is set by the degree of occupancy of the TF at the promoter independent of TF identity. This commonality across the TFs disappears when we measure the effect at −61, where the TFs exhibit different degrees of stabilization, with CpxR and AgaR engaging in significant stabilization of RNAP. To compliment this, we took CpxR and systematically quantified the contribution of the regulatory modes as a function of TF binding location and find that CpxR sets the degree of activation by engaging in two distinct regulatory paradigms. Binding locations that see strong activation have CpxR engaging in “coherent” regulation: the activation is enforced by stabilization and acceleration of RNAP. Locations with weak activation, however, have CpxR regulating the two modes oppositely by stabilizing RNAP but slowing the rate of promoter escape, demonstrating that such “incoherent” regulation plays a useful role by allowing a single TF to generate a spectrum of regulatory responses emerging from the relative effects of the TF on these distinct steps.

## RESULTS

### Thermodynamic model for single TF regulation

**Figure 1.**
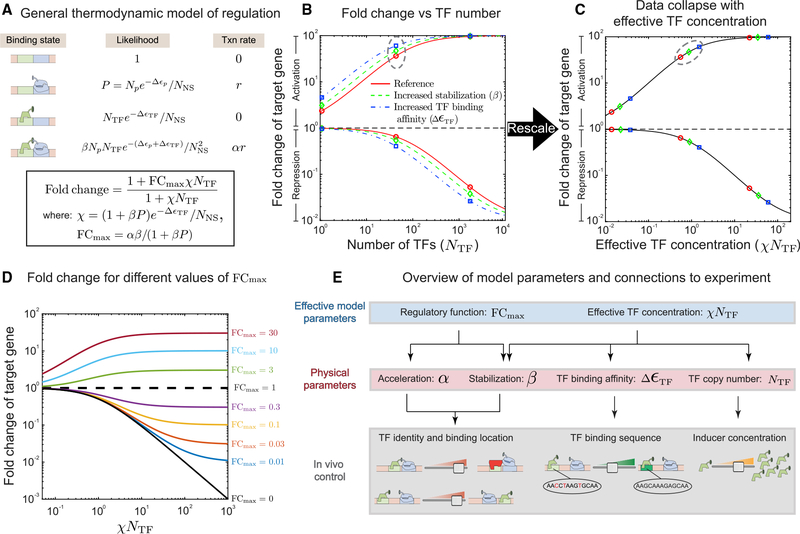
Thermodynamic modeling for measuring TF regulatory features (A) The thermodynamic model for the target gene allows for four states: unbound by TF or RNAP, bound by RNAP, bound by TF, or bound by both. The probability of each of these states occurring is listed in the center column. The rightmost column shows the transcription rates in these states. (B) Fold change versus TF copy number (*N*_TF_) for a gene regulated by an activator (top set of curves) or a repressor (bottom set of curves). The blue and green curves have the same FC_max_ as the red curve but with increased stability (β, green curve) or TF binding affinity (Δ*ε*_TF_, blue curve). (C) Replotting the curves in (B) as a function of effective TF concentration (*χN*_TF_) demonstrates that each of the curves now falls onto a single “collapsed” curve defined by the effective parameters FC_max_ and *χN*_TF_. (D) Data collapse curve for a range of different FC_max_ values. (E) The relationship between theoretical parameters of the model and *in vivo* molecular details of the regulation.

To deconvolve the role of TF copy number, binding affinity, and binding location from the intrinsic regulatory interactions of the TF with polymerase, we use a thermodynamic model of gene expression (Ackers et al., 1982; Kuhlman et al., 2007; Bintu et al., 2005; Kinney et al., 2010; Buchler et al., 2003; Vilar and Leibler, 2003; Garcia et al., 2012), where we consider only a single TF acting on an otherwise unregulated gene. Figure 1A shows the various promoter states considered in the model (left column), along with the relative probability of each state occurring (center column) and the rate of expression from each promoter state (right column); the promoter can be unbound by the TF and RNAP, bound by polymerase only, bound by the TF only, or bound by both. The probability with which these states occur is a function of each molecule’s (polymerase and TF) binding affinity with its specific DNA binding sites (Δε_P_ and Δε_*TF*_) and the available number of each molecule in the cell (*N*_*p*_ and *N*_*TF*_). For the co-bound state, we consider two distinct mechanistic influences of the TF on gene expression. The first effect represents altered stability of the polymerase at the promoter when TF is bound because of a favorable or disfavorable interaction between the TF and polymerase. As a result, the co-bound state occurs with increased relative probability to the single bound state by a factor β (implying an energetic interaction of *log*(*β*) in units of *k*_*B*_*T*). The second parameter α represents the change in transcription rate when the TF and polymerase are co-bound and is written as a multiplicative factor to the base expression rate of polymerase bound in the absence of the TF; for example, *α* = 2 would imply that the transcription initiation rate is doubled when the TF and polymerase are co-bound. In both cases the parameters represent increases in gene expression when greater than unity and decreases in gene expression when less than unity. Importantly, the parameters are not constrained and can, in principle, have opposing or compounding effects; i.e., this model allows a TF that stabilizes polymerase binding but slows the rate of transcription from that state, resulting in apparent activation or repression, depending on the relative strengths of those effects. The final parameter, *N*_*NS*_, is equated to the size of the genome in base pairs (4.6 × 10^6^) and is not varied in our experiments (for more details, see Phillips et al., 2019, and Bintu et al., 2005). Furthermore, the parameters related to polymerase binding can be simplified into a single parameter in our model as *P* = *N*_*p*_*exp*(−Δ*ε*_*P*_)/*N*_*NS*_.

The final expression, boxed in Figure 1A (and derived in the STAR Methods), predicts the fold change in gene expression of a target gene. Fold change is defined as the expression level of the target gene in the presence of a number of TFs (*N*_*TF*_) divided by the expression level in the absence of that TF (i.e., *N*_*TF*_ = 0). A fold change greater than 1 signifies activation, whereas a fold change below 1 signifies repression. The fold change equation is simplified by collecting the regulatory parameters into two effective parameters: *FC*_*max*_ and χ. *FC*_*max*_ represents the fold change when the number of TFs in the system are saturating; in the case of a repressor, it is the minimum fold change achievable, and in the case of an activator, it is the maximum fold change achievable. Importantly, *FC*_*max*_ depends only on a TF’s degree of acceleration (α) and stabilization (β) and not TF binding affinity or concentration. The second term, χ, represents the rate at which the fold change approaches *FC*_*max*_. This rate of approach depends on the TF binding affinity (Δ*ε*_*TF*_) and the degree to which the TF recruits/stabilizes RNAP (1 + *βP*). These factors, together with the number of TFs (*N*_*TF*_), can be thought of as an effective TF concentration.

These effective parameters are useful because they transform this system with many variables (TF and polymerase binding affinity, TF and polymerase number, degree of acceleration, degree of stabilization, etc.) capable of producing a diverse range of response curves in the fold change versus TF copy number space into a very simple system dictated by two fundamental quantities: the maximum fold change, *FC*_*max*_, and the effective TF concentration, *χN*_*TF*_. This is demonstrated in Figure 1B, where we plot fold change against TF number for an activator (top curves) and a repressor (bottom curves) with *FC*_*max*_ = 100 and *FC*_*max*_ = 1/100, respectively. In each scenario, we plot 3 colored curves. The red curve has no contribution from stabilization (*β* = 1). The blue curve is identical to the red curve except with slightly stronger TF binding affinity, and the green curve is again identical to the red curve except with significant contribution from stabilization (*β* = 10 with α adjusted to keep *FC*_*max*_ unchanged). Figure 1C demonstrates the fold change as a function of effective TF concentration, *χN*_*TF*_. When plotted this way, the data from all three curves collapses to a single curve that is determined entirely by the value of *FC*_*max*_, independent of specific values of α and β. Points with identical TF concentrations in Figure 1B now scatter on the collapsed curves, and the green and blue points (which had higher values of χ) are farther along the curve; a large value of χ hastens the approach to *FC*_*max*_ for the same TF concentration (*N*_*TF*_). Specifically, two TFs with similar net regulatory effect (similar *FC*_*max*_) that operate through different regulatory mechanisms (for instance, one through strong acceleration [large α, *β* ≈ 1] and one through strong stabilization [large β, *α* ≈ 1]) will trace out exactly the same curve in this space, but the strong stabilizer will have a higher effective TF concentration and, thus, move farther along the curve given the same TF concentrations and binding affinities. In Figure 1D, data collapse curves for a range of *FC*_*max*_ values are shown.

Figure 1E provides a hierarchical overview of model parameters and their relationship to controllable biological features of the *in vivo* system. The top level shows the effective parameters *FC*_*max*_ and *χN*_*TF*_, which define the contour of regulatory curves like those in Figure 1D. These effective parameters are composed of a combination of the “physical” parameters on the second level of the diagram. These parameters correspond to basic features of the system, such as numbers of molecules, affinities, or interactions between molecules. The third level of the diagram shows the biological controls we have available to control the corresponding physical parameters. The approach we take below will be to profile the regulatory function and characterize the inherent regulatory parameters (α and β) of six TFs (AgaR, ArsR, AcrR, AscG, BetI, and CpxR) by controlling the copy number, binding location, and binding sequence of each. A potential challenge in determining α and β stems from their connectedness in the effective parameters χ and *FC*_*max*_. Because χ is proportional to (1 + *βP*), if the stabilization parameter β is much smaller than 1/*P* ≈ 15 (measured previously for the promoter sequence used in our experiments; Brewster et al., 2012), then χ will no longer strongly depend on β because (1 + *βP*) ≈ 1. In cases such as this, which we label “weak stabilization,” we are left with only one effective parameter, *FC*_*max*_, to determine the two regulatory parameters α and β, and it is not possible to distinguish between regulation driven by a change in the transcription rate (acceleration/deceleration) or by modulation of polymerase occupancy at the promoter (stabilization/destabilization).

### Experimental measurements of individual TF regulatory function

**Figure 2.**
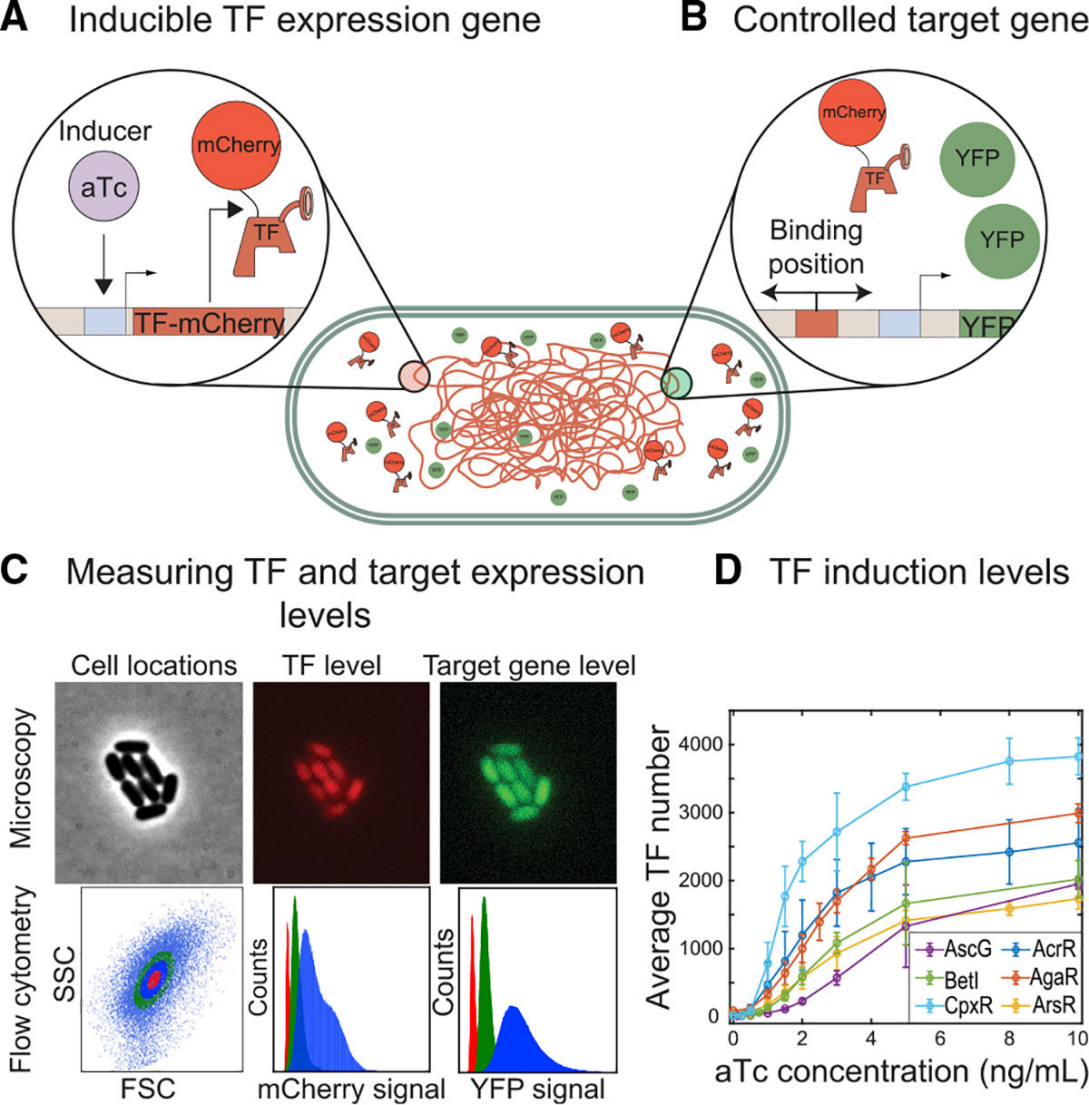
Experimental setup for measuring the TF position-dependent regulatory profile (A) The inducible TF expression strains consist of a set of base strains where the endogenous copy of any one of our 6 TFs is knocked out and reintroduced as a TF-mCherry fusion at the *ybcN* locus expressed from an inducible *tet* promoter. (B) Regulation by the controlled TF is measured using a synthetic target promoter driving YFP expression integrated to the *galK* locus. The target promoter is designed to be unregulated except by a single binding site for the controlled TF. The sequence and location of this binding site can be controlled systematically. (C) The quantitative regulation is measured as the fold change in YFP expression as a function of mCherry signal. (D) The range of TF concentrations explored for each TF is shown. Data points for each TF represent the mean number of TFs across 3 replicate measurements (*n* = 3), with the error bars representing the standard error of the mean. Here we converted the arbitrary mCherry fluorescence signal into number of TFs using a fluctuation counting method detailed further in the STAR Methods.

To measure regulation by an individual TF in *E. coli* as a function of TF identity and binding position on the promoter, we utilize synthetic techniques to create simple and controllable gene circuits. In this approach, we have created *E. coli* strains, illustrated schematically in Figures 2A and 2B, where the endogenous copy of each studied TF is knocked out and reintroduced as a TF-mCherry fusion integrated into the genome at the *ybcN* locus (Figure 2A). Expression of the synthetic TF-mCherry promoter can be induced with anhydrotetracycline (aTc) (for details, see STAR Methods). This system enables precise control of TF copy number (Figure 2D), which is measured by wide-field fluorescence microscopy or flow cytometry (Figure 2C; STAR Methods). In our data, we use a fluctuation-counting method to convert the arbitrary fluorescence from the microscope into TF copy number. We convert the arbitrary fluorescence from the cytometer to TF number through a reference measurement on the microscope measured in parallel on the flow cytometer. This process has been described previously (Rosenfeld et al., 2007; Teng et al., 2010; Brewster et al., 2014) and in more detail in the STAR Methods. Using this system, we are able to induce these TFs from leaky levels (several per cell) up to several thousand per cell; the full induction curve of each strain is shown in Figure 2D. Importantly, although we control the concentration of the TF, many TFs are capable of existing in distinct binding conformations that may alter the active TF number, the regulatory parameters (α and β), or both. For all but one of the TFs studied here, we expect that the TFs will always be active under our growth conditions; however, BetI is inactivated by choline (which we do not control for), and therefore we expect to have some fraction of BetI inactive at low TF concentrations (Lamark et al., 1991; Gama-Castro et al., 2016).

In each of these individually tunable *tf-mCherry* strains, a target promoter that drives YFP expression is integrated into the genome at the *galK* locus (Figure 2B). The basic promoter incorporates a modified *lac* RNAP binding sequence where the RNAP occupancy term, *P*, was measured previously (Brewster et al., 2012). Otherwise, the promoter is designed to be free of specific known TF binding sequences. To study the regulatory role of a specific TF, we introduce a TF-specific binding site (chosen from an array of known binding sites with strong evidence of that particular TF binding (Salgado et al., 2013; Keseler et al., 2017)) cloned directly downstream of the transcription start site (TSS), annotated as +1 for simplification, or centered at 61 bases upstream of the TSS, annotated as −61. The effect of TF binding to the promoter is then measured in terms of YFP fluorescence protein expression as a function of average number of TFs per cell for a given induction condition (Figure 2C). For this work, our focus is to select a binding site that will bind the TF. The affinity of the site or how the particular choice may influence the regulatory parameters is not something we explore exhaustively here. The specific binding sites chosen for each TF can be found in the STAR Methods.

### Regulatory response of six different TFs at +1 and −61

**Figure 3.**
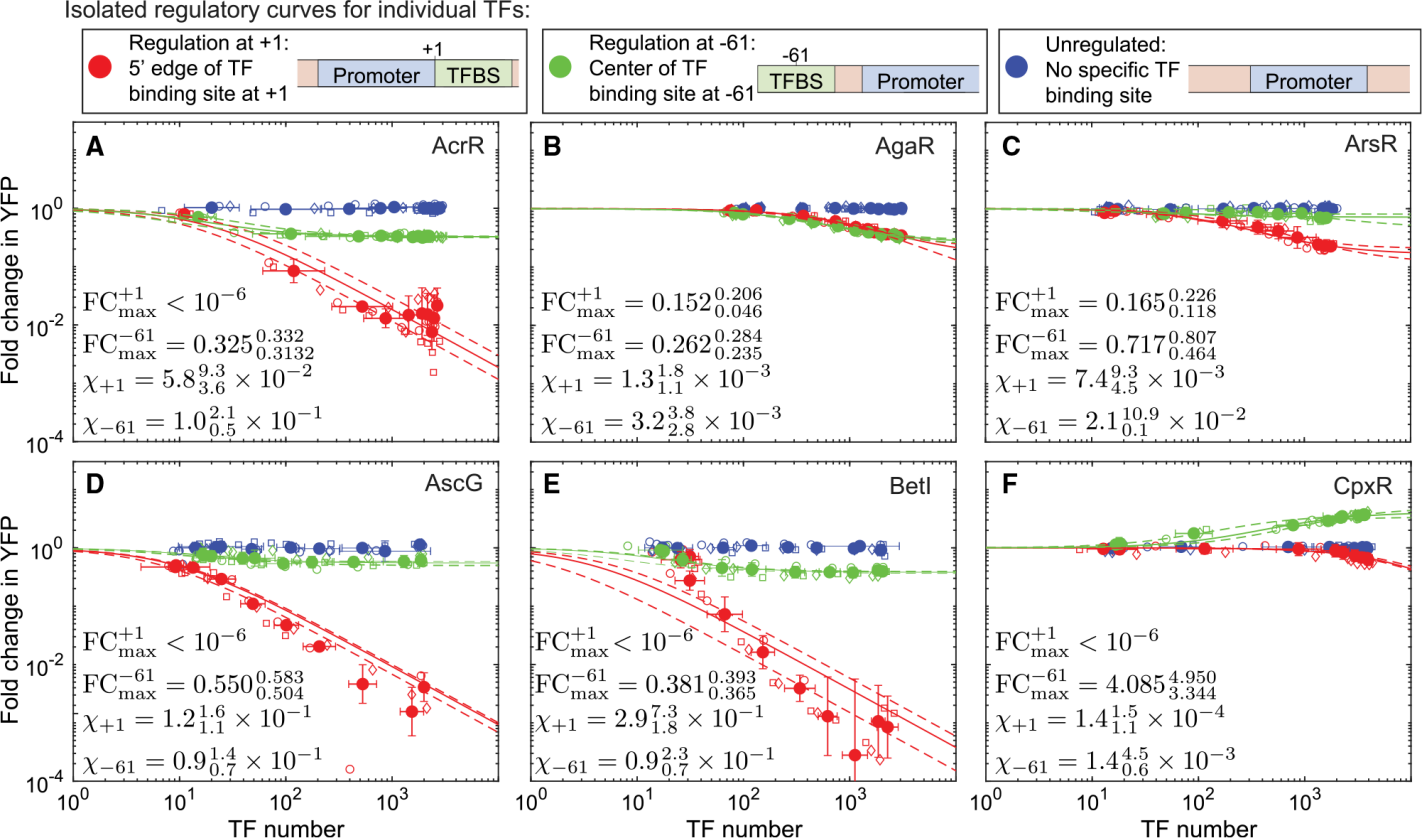
Regulatory curves for individual TFs (A–F) Each curve shows the response of a gene regulated only by the controlled TF to a measured level of TF. In all cases, the average number of TFs from each induction condition is found by converting the arbitrary fluorescence signal of each TF-mCherry fusion to TF number through a fluctuation counting method. For the “no binding site” control data (blue points), the fold change is typically 1 for all TF concentrations; in other words, there is no regulatory response to the TF in the absence of a binding site. The empty data points on the plots represent the sample means of TF number and fold change in each of three replicates. The filled data points represent the mean of the 3 replicates, and the error bars represent the standard error of the mean. When the binding site is inserted just downstream at +1 (red points), the observed regulatory function is always repression. However, at −61 (green points), the response can vary between repression that is as strong as +1 (i.e., AgaR in B), repressive but weaker than at +1 (i.e., AcrR or BetI in A and E), or it can have the opposite role and activate (i.e., CpxR in F). The fits represent least-square optimization of the theory presented in Figure 1, with dashed lines representing the 95% confidence interval generated by bootstrap sampling.

**Figure 4.**
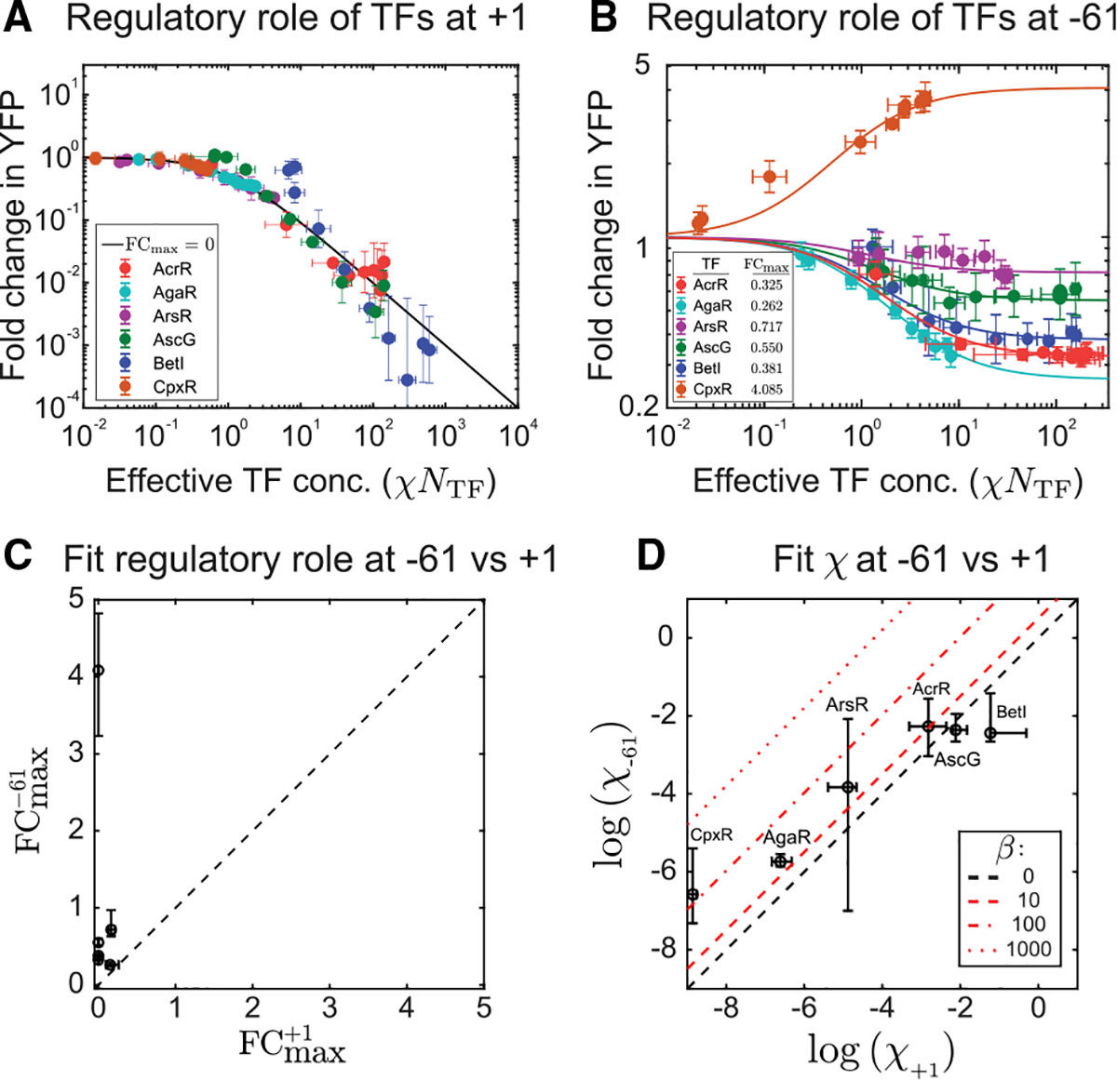
Fold change for a given regulatory location versus *N*_eff_ (A and B) The regulatory curve for all TFs when acting at (A) +1 and (B) −61 plotted against *N*_eff_ = *χN*_TF_. Filled data points for each TF along with the error bars represent the mean and standard error of the sampling mean as in Figure 3. In all cases, the binding energy and FC_max_ are determined from fitting the equation in Figure 1A to the +1 and −61 data independently. Although the data for +1 are well described by a single regulatory behavior for every TF (pure repression; i.e., FC_max_ ≈ 0), the same TFs at −61 have a spectrum of quantitatively distinct regulatory behaviors. (C) There is no correlation for the overall regulatory role of the TF between +1 and −61, indicating position dependence for the regulatory role of these TFs. (D) The inferred TF binding affinity is consistent between +1 and −61 for all but two TFs corresponding to AgaR and CpxR, possibly indicating a contribution from TF stabilization (*Pβ* > 1).

Figures 3A–3F show the fold change in YFP expression (promoter activity) as a function of TF copy number for the TFs examined in this study, measured using single-cell fluorescence microscopy. In these plots, regulation at +1 is shown as red points, regulation at −61 is shown as green points, and a control promoter with no TF binding site is shown in blue. The six TFs display diverse regulatory behavior that depends on the TF identity and TF binding location on the gene. For example, CpxR (Figure 3F) is a repressor when bound at +1 but activates at −61. AscG (Figure 3D) is a strong repressor at +1 but has almost no regulatory role at −61, and BetI (Figure 3E) represses at +1 and −61 but much more weakly at −61 despite binding to the same sequence at both locations. One commonality between the curves is that, at +1, every TF in this study acts as a repressor. Naively, the “strength” of this repression appears rather diverse; the repression from some TFs reduces YFP expression by 10^−3^ in fold change (BetI), whereas others never drop below 10^−1^ (AgaR). The solid lines in this figure show a fit to the model in Figure 1A (boxed equation). In this case, we are explicitly measuring the number of TFs, *N*_*TF*_, and we fit for the two unknown parameters χ and *FC*_*max*_ (Figure 1A). In four of the six curves (Figures 3A and 3D–3F), the +1 regulation data give *FC*_*max*_ consistent with zero. This result is consistent with the regulatory mode of perfect repression; i.e., that the TF completely shuts off the gene when bound. Therefore, the difference in regulation at +1 between the TFs must be attributed entirely to binding affinity or differing levels of stabilization (β) between TFs. A typical assumption is that TFs operating at +1 regulate by steric hindrance (*β* = 0) so that, when the TF is bound at +1, polymerase binding is occluded. Previous studies (Ptashne and Gann, 1998; Ackers et al., 1982; Brewster et al., 2014; Forcier et al., 2018) support this assumption. The remaining curves (Figures 3B and 3C) show *FC*_*max*_ of order 10^−1^, but in both cases, the binding is weak to the point where we do not see the expected saturation of fold change with TF copy number, and, therefore, the data from these curves are also consistent with *FC*_*max*_ of zero with a slightly increased value of χ to compensate. The collapse of all +1 regulation data to the perfect repression contour (*FC*_*max*_ = 0) is demonstrated in Figure 4A, where we plot the fold change against the effective TF concentration, *χN*_*TF*_, for these six TFs; the +1 data for all six TFs largely fit to a single regulatory contour associated with perfect repression when the extrinsic features, such as TF copy number and binding affinity, are “normalized away.”

Although, at +1, the fold change curve for each TF collapsed on a unifying regulatory profile (with *FC*_*max*_ = 0), at −61, these TFs operate with a diverse range of regulatory effects; some TFs mirror the function at +1, showing a profile similar to the response function at +1, whereas others show limited repressive capabilities that saturate at specific fold change values (*FC*_*max*_), and still other TFs activate expression at −61. To quantify *FC*_*max*_ and χ for each TF acting at −61, we fit the data in Figure 3 with the theory in the fold change equation above. We find that the repressive TFs have *FC*_*max*_ values ranging between 0.2 and 0.7, whereas the lone activating TF is around 4. In Figure 4B, we plot the fold change data against the effective TF concentration, *χN*_*TF*_. Now, rather than each TF following the same trajectory, the regulation data for these six TFs follow unique trajectories corresponding to specific values of *FC*_*max*_. Figure 4C demonstrates that *FC*_*max*_ at +1 and −61 for these six TFs is not correlated between the two locations.

In Figure 4D, we show the fit value of χ for each TF at −61 against the fit value of χ at +1. Recall that χ is composed of the product of two effects: the binding affinity and the stabilization. We do not expect the TF binding energy, Δ*ε*_*TF*_, to depend on where the binding site is located because the binding sequence is not changed (Salgado et al., 2013). The other possible contribution to χ comes from stabilization and takes the form (1 + *βP*), which means that β must be of order 1/*P* or larger for stabilization to affect χ. We expect that the data points will fall into two potential outcomes. Points that lie on the black dashed line indicate that stabilization does not play a significant role in the regulation at −61. However, points that are above the black dashed lines imply that the TF utilizes stabilization of RNAP (shown as red lines in Figure 4D). Many of the data points are consistent with small or zero β, but two TFs, corresponding to AgaR and CpxR, are significantly above this line, implying that stabilization may play a role in their respective regulation. The implied magnitude of stabilization is shown by the red lines. Interestingly, AgaR in this case is a repressor but appears to impart strong stabilization, suggesting that, even though this TF stabilizes (*β* > 1) the polymerase at the promoter, it more strongly decreases the rate of transcription from polymerase bound at the promoter (*α* < 1), resulting in a net repression of gene expression. This highlights a mechanism of repression that is fundamentally distinct from the downstream (+1) position regulation for AgaR and demonstrates an incoherent regulatory strategy where the TF engages RNAP at two distinct steps with opposing effects.

### Profiling the spatial regulatory landscape of the CpxR TF

**Figure 5.**
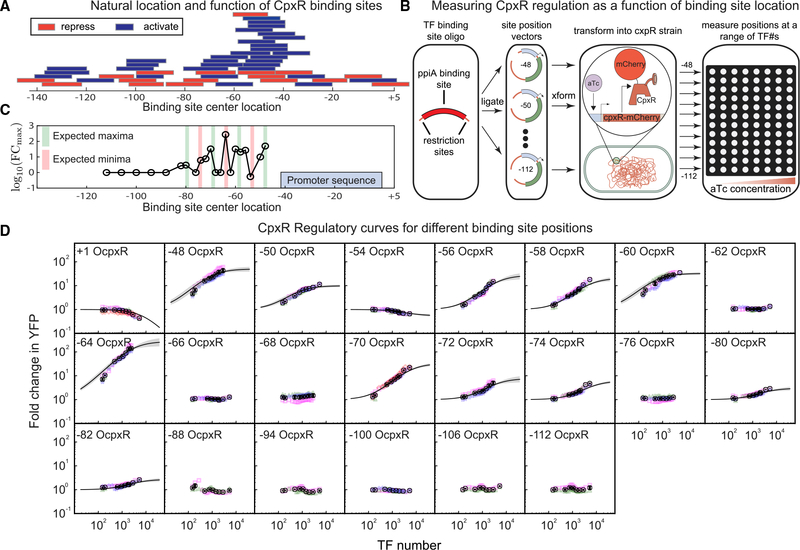
Position-specific regulatory profiles of the CpxR TF (A) The distribution of CpxR binding sites across all naturally occurring genes in *E. coli*. The length of the rectangles represents the span of the binding sequence, and the border color represents activation (blue) or repression (red). The majority of the binding sites are centered between the −40 to −80 positions. (B) Schematic of the strategy for constructing and measuring CpxR acting at a specified binding site (the *ppiA* binding sequence) inserted at 21 upstream positions and 1 downstream position on the promoter. (C) The mean of the inferred maximal fold change (*FC*_*max*_) for each of the 21 upstream binding locations as a function of the binding location at the promoter. The centers of the red and green shaded areas denote the presumed locations for the minima and maxima of the regulatory response (anchored on −48) based on the 10.5-bp periodicity of B-form DNA. (D) The regulatory profile of CpxR as a function of TF copy number for the 22 binding locations. Each panel shows the TF copy number on the x axis and the fold change in YFP on the y axis of individual replicates (colored points). For all positions that show regulation (plots with the model curves), n≥_3, with n as the number of replicates. The black points represent the mean and standard error of these replicates. For convenience, this is not shown for every TF number in the plot. The dashed line running though the data points is the theory prediction based on inference of the model parameters detailed in Figure 6. The shaded regions represent ± 2 standard deviations of the thermodynamic model (Figure 1A) conditioned on the inferred model parameters.

We now examine how the regulatory parameters that quantify stabilization (β) and acceleration (α) vary with binding location for one TF, CpxR. As demonstrated in Figure 5A, CpxR naturally binds to a wide range of promoter locations to regulate dozens of different genes in *E. coli*. However, the regulatory role of CpxR as a function of binding location is unclear from these data; repressive and activating interactions are attributed to many of the locations upstream of the promoter. Furthermore, we have evidence that CpxR is capable of regulating through stabilization at −61, implying that we may be able to separate regulation through stabilization and regulation through acceleration in a more thorough examination. To measure the isolated regulatory behavior of CpxR as a function of binding location, we take the same synthetic target gene (Figure 2B) and move the TF binding site between positions centered at −48 bp from the TSS to −112 bp from the TSS (Figure 5B). We chose this range because the vast majority of natural CpxR binding sites occur within these limits (Figure 5A). To enable rapid cloning and measurement, the target gene is cloned into a low-copy plasmid (rather than integrated into the genome), and fold change in target expression and TF abundance is measured using flow cytometry (rather than single-cell microscopy) (Table 1). We find consistent results with the target gene on the plasmid or integrated into the genome (STAR Methods).

The data for fold change as a function of CpxR copy number for the binding location sweep is shown in Figure 5D. Regulation of the positions studied here is primarily activation, with 11 positions showing increased expression ranging from 2-fold to over 100-fold and just one upstream position showing moderate repression. We find that the regulatory effect of CpxR depends strongly on binding site location; the −54 binding location shows weak repression but is flanked by activating positions only a few bases away (−50 and −56). As expected, positions that are far from the promoter (in this case, beyond roughly 82 bp) show little to no regulation. In Figure 5C, we show fit values of *FC*_*max*_ as a function of binding location on the promoter for these data. We expected to see an 11-base periodicity in *FC*_*max*_ corresponding to the helicity of DNA (Müller et al., 1996; Garcia et al., 2012; Sharon et al., 2012); we see this very roughly with maxima in activation around −48, −60, −70, and −80 (± 1 bp), but −64 is a strong outlier that is expected to be close to a minimum but is strongly activating as measured here.

**Figure 6.**
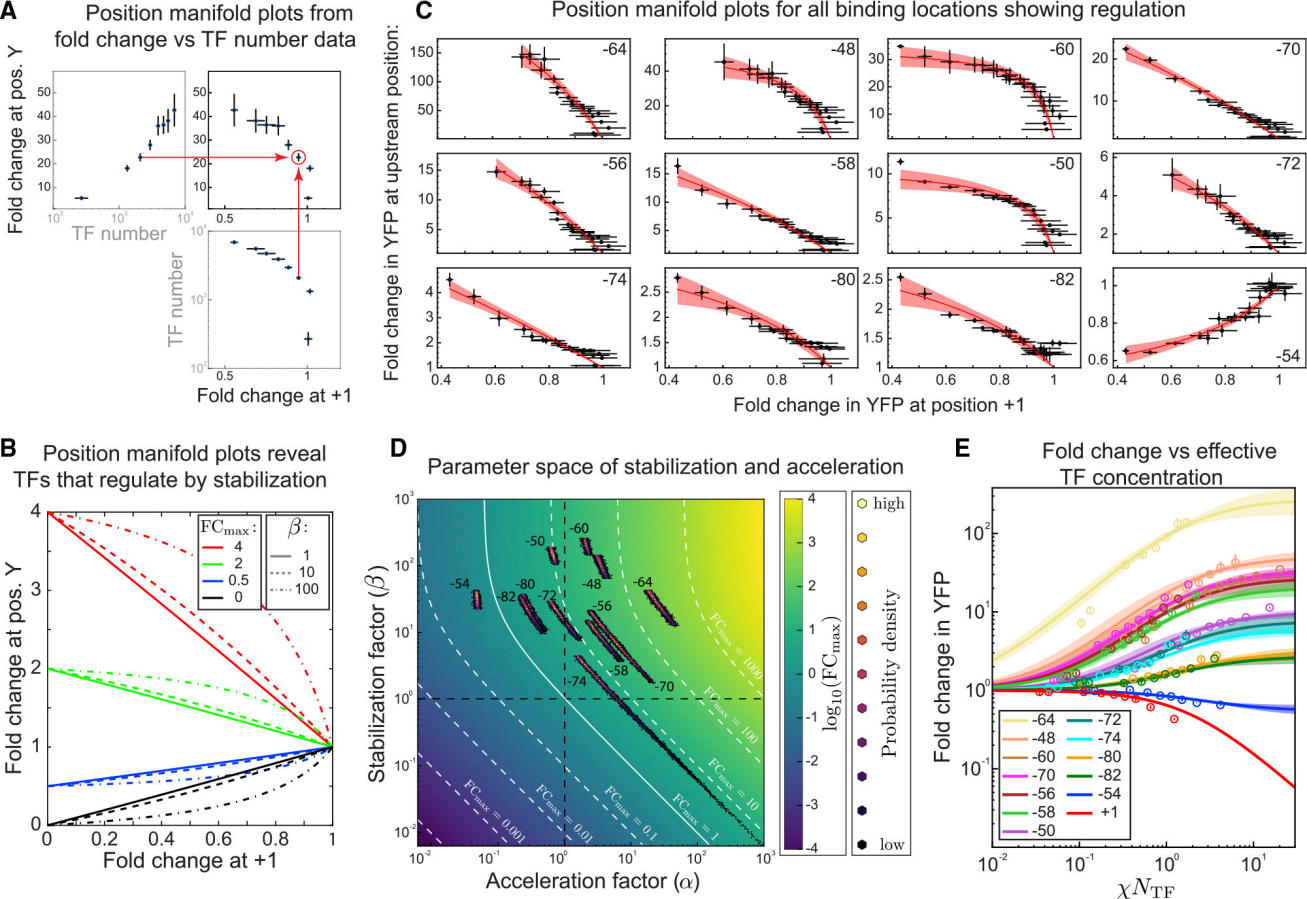
CpxR binding location determines the mode of TF-RNAP regulation (A) Tracing out the position regulatory manifold using TF abundance from two different binding locations. (B) The concentration manifold curves are predicted to be straight lines when β is small but curved for larger values of β. (C) Concentration manifold plots for the 12 upstream regulatory positions as a function of the fold change at +1. The solid red line represents the mean of the model regulatory profile (thermodynamic model) generated from the inferred acceleration and stabilization parameters, and the shaded regions represent ± 2 standard deviations from the mean. Data points represent the mean and standard error of the fold change as seen in the plot of Figure 5D for each respective position. (D) Phase plot of *FC*_*max*_ as a function of α and β parameters. The horizontal black line marks *β* = 1 (no stabilization or destabilization), and the vertical black dashed line marks *α* = 1 (no acceleration or deceleration). The white lines represent contours of constant *FC*_*max*_. The colored points represent the parameter inference of α and β for each of the regulatory positions. The median as well as the 68% credible intervals of the inferred parameters for each position are reported in Table 2. (E) Plot of fold change against the effective TF concentration *χN*_*TF*_ for the 13 regulatory positions (12 upstream and 1 downstream) using parameters derived from the concentration manifold plots in (C).

To precisely extract the regulatory parameters α and β from these data, it is helpful to replot the fold change data using a manifold approach. A recent approach used regulatory manifolds to explore how the CRP TF acted on dozens of different promoters and employed different RNAP binding sequences to trace out the regulatory space of the TF (Forcier et al., 2018). In this case, the power in the manifold approach arises from the ability to eliminate the need to measure (or know) the binding affinity of polymerase to each promoter; by replotting the data, specific data trajectories (or “allelic manifolds”) corresponded to unique values of CRP regulatory parameters. For our study, we use a “concentration manifold” to avoid the need to measure the effective concentration (in particular, the binding affinity of the TF). This approach is demonstrated schematically in Figure 6A. The concentration manifold plots the fold change at one position against the fold change of another position; each data point in this space corresponds to a measurement of both TF binding positions at the same effective TF concentration. In this case, we chose to plot all position data against the corresponding fold change at +1; we chose +1 because, based on Figure 4A, we believe that regulation is “pure steric hindrance” (*FC*_*max*_ = 0, *β* = 0) here. The advantage of this approach is that it enables inference of β at other positions based on the curvature of the data without the need to simultaneously infer the TF binding affinity or TF copy number (see STAR Methods for model assumptions). As seen in Figure 6B, data in the concentration manifold are expected to be lines emanating from (1, 1), which is the defined fold change of both locations when the number of TFs is zero. As TF concentration is increased, the fold change at +1 decreases toward 0, and the fold change at the second position will increase or decrease depending on the regulatory function at that position; therefore, activating positions have curves that rise as you move toward zero on the x axis, whereas repressive positions decrease. The role of β is clear in this formulation. When β is small (compared with 1/*P*), the profiles are straight lines. However, larger β will cause the curve to rise or fall more rapidly than linear, reaching *FC*_*max*_ at higher values of the corresponding +1 Fold-change.

In Figure 6C, we replot the data from Figure 5D as fold change at an upstream regulatory position against fold change at +1; each data point represents a measurement of fold change at these two different binding locations for a given TF copy number within the cell. The plots are arranged from the highest *FC*_*max*_ (strong activation at −64) to the lowest *FC*_*max*_ (weak repression at −54). The solid line represents our model curve using the inferred values of α and β. Based on the inferred stabilization values, we find that the activation profiles across the position sweep is driven by varying degrees of stabilization and acceleration. The inferred values for α and β of all measured positions are shown in Table 2. We find strong stabilization in regulation at positions −50, −60, −54, −48, and −64; Figure 6C shows the curvature we expected to see from strong stabilization. Several positions (−58, −56, −70, and −74) have regulation profiles that are approximately straight lines, implying that CpxR destabilizes or regulates through weak stabilization, i.e., (1 + *βP*) ≈ 1.

Figure 6D shows a heatmap for *log*(*FC*_*max*_) as a function of the regulation parameters α and β. The dashed black lines, which denote α and β equal to 1, divide the map into four quadrants, each quadrant with a specific qualitative regulatory scheme. The top right and bottom left quadrants represent coherent regulation strategies where α and β contribute to activate (top right) or repress (bottom left) gene expression. On the other hand, the top left and bottom right quadrant are incoherent in the sense that α and β have opposing regulatory effects: TFs in these quadrant slow the initiation rate of transcription while stabilizing polymerase at the promoter (top left) or increase the initiation rate of transcription while destabilizing the polymerases’ presence at the promoter (bottom right). The solid white contour in this plot shows where these two effects balance and the net fold change is 1; left of this line represents TFs that repress, and right of this line represents TFs that activate. Contours of constant *FC*_*max*_ are drawn as white dashed lines, marking 10-fold increases/decreases in *FC*_*max*_. On this plot, we also show the inferred probability distribution of the parameters α and β for each position in our data. The black points are lower probability, with lighter points representing higher probability values for the α and β parameters. One notable phenomenon is that, for positions with β less than roughly 10, the inference begins to fail for α and β. This results in inference clouds with “tails” that stretch across quadrants and precludes assessment of the mode of regulation (see position −74). The alignment between the inference clouds of these positions and the constant *FC*_*max*_ contours, however, shows that, although we make very precise estimations of *FC*_*max*_, the values of α and β are less certain and correlated. This is an unfortunate consequence of the weak stabilization limit in our model resulting in parameter combinations that encompass coherent and incoherent regulatory regimes, which could explain the data well (Transtrum et al., 2015).

At some positions (−54, −50, −80, and −82), we see the incoherent behavior discussed above, where the TF stabilizes polymerase at the promoter and also slows the rate of initiation, essentially serving opposing functions in influencing gene expression. For −54, the net effect of these opposing mechanisms is repression, whereas at −80, −82, and −50, the result is activation. However, the positions with strong activation signatures (−48, −60, and −64) as well as some intermediate ones (−56, −58, and −70) have stabilization and acceleration values that impart a coherent strategy of regulation where RNAP recruitment and acceleration of transcription work together. Interestingly, all but one of the regulatory positions studied here show clear positive stabilization (*β* > 1), even the lone repressive position (−54). In our data, upstream regulation by CpxR typically involves stabilizing RNAP, regardless of the net regulatory function (repression or activation). However, the level of acceleration/deceleration varies more significantly between positions from a roughly 20-fold deceleration, which results in overall repression of expression, up to a 25-fold acceleration, which results in strong activation.

Finally, combining the inferred regulatory parameters, α and β, determined through inference in the concentration manifold space, with the measured extrinsic features (TF copy number and binding affinity) of gene regulation produces model curves using the effective parameterization that fit our data well. The effective parameters *FC*_*max*_ and χ for the CpxR TF data (see STAR Methods for details) are used to plot the fold change as a function of *χN*_*TF*_ for the 13 regulatory positions in Figure 6E. Crucially, we demonstrate that the two key variables, χ and *FC*_*max*_, are effective in capturing the fold change position sweep profiles similar to the plots in Figures 4A and 4B.

## DISCUSSION

To build a predictive understanding of gene regulation, we need to understand not just where and when TFs bind but also learn the function and magnitude of the mechanisms of regulation at work by each TF (Weingarten-Gabbay and Segal, 2014; Gertz et al., 2009; Bylino et al., 2020). Often, it is difficult to separate the contributing factors of regulation, such as the TF binding affinity, copy number, and interactions with other TFs, from the regulatory role of the TF that is characterized by its interactions with RNAP at the promoter (Ross and Gourse, 2009). Here we use a synthetic biology approach to measure the isolated regulatory effect of a TF on an otherwise constitutive promoter. These data are interpreted through a thermodynamic model of gene expression that treats the regulatory role of TFs as a combination of interactions that stabilize (or destabilize) the polymerase at the promoter and interactions that accelerate (or decelerate) the rate of transcription when the TF is cobound with polymerase. The model used here allows both modes simultaneously and, importantly, enables us to quantify TF regulatory function continuously rather than categorically as “activators” or “repressors.” Using this model, we are able to characterize the wide range of regulation we see from the TFs in this study, which ranges from 10,000-fold repression up to 100-fold activation and everything in between with the same model. We believe that this fluid classification of TF function can be a useful tool for characterizing TFs for the purpose of model building and predictive design of gene regulation.

We found that, for TFs operating immediately downstream of the promoter, the regulation of each TF was consistent with strong repression (i.e., with *FC*_*max*_ ≈ 0). Despite the large range in magnitude of regulation at this location, the same intrinsic regulatory mechanism seems to be conserved; differences in the magnitude of regulation were primarily due to differences in TF binding affinities rather than in the fundamental regulatory mechanisms of the TFs. In contrast, when these same TFs bind 61 bp upstream of the promoter, the regulatory function of the TFs varied more substantially. We find that some TFs remained strong repressors (similar to their function at +1), but other TFs only weakly repress expression regardless of TF copy number. We attribute this to intrinsic properties of the TF, polymerase (de)stabilization and (de)acceleration of transcription initiation by the TF, which depend on TF identity. Furthermore, when profiling the regulation of CpxR at upstream binding locations, we find that this TF can regulate multiple steps of the transcriptional process and joins a growing body of evidence for TFs engaging in complex regulatory maneuvers at the promoter (Smith et al., 1996; Monsalve et al., 1997; Jensen et al., 2019). This insight into how activation is actually brought about by the independent contributions of acceleration and stabilization demonstrates the applicability of the model to *in vivo* data and complements previous work investigating the regulatory profile of the CRP TF (Forcier et al., 2018) as well as *in vitro* biochemical and structural considerations probing the kinetics of activation (Rhodius et al., 1997). We find that the contributions of these two mechanisms do not correlate with position; in some locations, we found stabilization and acceleration acting together to produce strong activation, and in other locations, deceleration and stabilization worked incoherently, resulting in weaker activation and repression. A startling feature of this incoherent regulation was its presence in the regulatory response of an activator (CpxR) and a repressor (AgaR) and demonstrates that this type of regulation is not just accessible to TFs but may be a pervasive aspect of TF-RNAP regulation.

The concept of stabilization and acceleration working antagonistically, with the step carrying the larger effect size ultimately determining the status of expression (activation or repression), has implications for the current paradigm of viewing TF regulation, particularly activation in the context of class I and class II promoters. This delineation of promoter class is based on the type of molecular contacts the activator makes with RNAP (Li et al., 1997; Niu et al., 1996; Lee et al., 2012; Zhou et al., 2014; Savery et al., 2002). For specific TFs, mapping between contacts and regulatory mode has been established (Li et al., 1997; Savery et al., 2002) However, addressing how these contacts shape the relative effects of α and β across a wide range of TFs would fill a vital gap in elucidating the molecular determinants that give rise to coherent and incoherent regulatory regimens. Such information would allow more complete characterization of TFs, and, in conjunction with methods of profiling TFs through genome-wide occupancy techniques, provides an edge in the challenge of uncovering an “expression code”: i.e., a set of rules that govern the magnitude and duration of gene expression from natural promoters (Kinkhabwala and Guet, 2008; Beer and Tavazoie, 2004; Ireland et al., 2020). Realizing this goal requires systematic characterization of position-dependent TF regulatory profiles to determine the spatial landscape of α and β for TFs across different families, with the aim of generating a “regulatory compendium” that classifies TFs according to their regulatory mode. This argument, however, is predicated on our ability to infer regulation driven by stabilization and acceleration with sufficient precision. One unfortunate feature of our experiments, as designed, is our inability to measure destabilization with good precision (*β* ≤ 1) and limits the characterization of TFs that engage through this mode. In these experiments, FCmax acts as a “stiff” parameter that completely determines the expected regulatory outcome, whereas the individual values of α and β are “sloppy” (Machta et al., 2013; Transtrum et al., 2015). One potential way to overcome this is to select a stronger promoter sequence for the target gene. The selected sequence we used in our synthetic circuit was an attenuated form of the *lacUV5* promoter, which was selected to increase the dynamic range of the promoter. Therefore, measuring the isolated regulatory function of TFs may require a range of promoters to fully characterize the wide array of possible regulatory behaviors.

Here we focus entirely on the isolated regulatory role of each TF, but it is clear that one of the next steps is to probe how quantified TFs regulate together. Extending the simple thermodynamic model to incorporate regulation directed by multiple TFs will play a crucial role in untangling elaborate regulatory architectures (Buchler et al., 2003), especially those found in eukaryotes. Indeed, thermodynamic models have been employed to interrogate the regulatory function of eukaryotic promoters involved in key processes such as cellular differentiation, body patterning, and a host of other biological roles (Segal et al., 2006, 2008; Sayal et al., 2016; Chen et al., 2008; Ay and Arnosti, 2011; Fakhouri et al., 2010; Bashor et al., 2019). The ability to distinguish and quantify the different modes of regulation (stabilization and acceleration) and characterize TFs based on them is important for developing general theories of regulation that include multiple TFs that act on different kinetic steps of the transcription process (Scholes et al., 2017; Martinez-Corral et al., 2020; Wong and Gunawardena, 2020); predictions for the combined regulatory effect of two stabilizing TFs should be different than predictions for a stabilizing TF acting together with an accelerating TF (Scholes et al., 2017). With each characterized TF, we can develop an empirical baseline or null hypothesis for what a TF should do on a gene; departures from this expectation, because of emergence of complex regulatory phenomenon brought about by TF-TF interactions (Weingarten-Gabbay and Segal, 2014), allosteric interactions (Rosenblum et al., 2020; Kim et al., 2013), or other effects indicate surprises that warrant testing in these expanded models.

## STAR★METHODS

### RESOURCE AVAILABILITY

#### Lead contact

All request for information regarding datasets, materials, and reagents as well as questions pertaining to the manuscript should be directed to Robert Brewster (Robert.Brewster@umassmed.edu).

#### Materials availability

All *E. coli* strains and plasmids generated in this study are available on request by contacting the lead contact.

#### Data and code availability

- All data reported in this paper will be shared by the lead contact upon request.
- This paper does not report original code.
- Any additional information required to reanalyze the data reported in this paper is available from the lead contact upon request.

### EXPERIMENTAL MODEL AND SUBJECT DETAILS

#### Microbial strain and culture conditions

*E. coli* strain MG1655 was the base strain used for all synthetic regulatory circuit strains constructed and measured in this work. For a comprehensive list of engineered strains, see Tables S2 and S4. All strains were cultured at 37° C with 250rpm in a shaking incubator in an initial culture of LB and appropriate antibiotics until saturation. Cultures were then diluted 10^4^- to 10^5^-fold into 1 mL of fresh M9 minimal media supplemented with 0:5 percent of glucose at different aTc concentrations. We then assessed when the cultures were at steady state using OD measurements, and performed our quantitative measurements of TF copy number and YFP expression on either microscopy or flow cytometry immediately. More details on these procedures can be found in Culture conditions and Data acquisition procedures for Microscopy and Flow Cytometry Data in the Method details. The comprehensize list of engineered strains are found in Tables S2 and S4.

### METHOD DETAILS

#### Thermodynamic model for single TF regulation

In the work here we use the standard form of the thermodynamic model as derived elsewhere (Ackers et al., 1982; Bintu et al., 2005; Buchler et al., 2003). In our framework, we include an additional consideration of the TF altering the rate of transcription as detailed in Garcia et al. (2012). The partition function for this system is:

(Equation 1) $$Z=\left(1+\left(N_{p}/N_{NS}\right)e^{-\left(\Delta \varepsilon_{p}\right)}+\left(N_{TF}/N_{NS}\right)e^{-\left(\Delta \varepsilon_{TF}\right)}+\left(N_{p}N_{TF}/N_{NS}^{2}\right)e^{-\left(\Delta \varepsilon_{p}+\Delta \varepsilon_{TF}+\Delta \varepsilon_{l}\right)}\right),$$

where each term (in order) represents the weight of the unbound, bound by polymerase, bound by TF and cobound state. The terms *N*_*p*_ and *N*_*TF*_ are the total number of polymerase or TF molecules, with the term *N*_*NS*_ in the denominator scaling the respective terms with the total number of potential binding sites on the genome to give an effective concentration on the chromosome. The energy terms Δ*ε*_*p*_ and Δ*ε*_*TF*_ are the binding affinities of polymerase or TF to their promoter or operator site. The stabilization term (β) as discussed in Figure 1 is represented by the exponentiation of Δ*ε*_*I*_. The probability to find polymerase bound as a function of TF number is then,

(Equation 2) $$P_{bound\;}\left(N_{TF}\right)=\frac{\left(\left(N_{p}/N_{NS}\right)e^{-\left(\Delta \varepsilon_{p}\right)}+\left(N_{p}N_{TF}/N_{NS}^{2}\right)e^{-\left(\Delta \varepsilon_{p}+\Delta \varepsilon_{TF}+\Delta \varepsilon_{l}\right)}\right)}{Z}.$$

To compare with experimental measurements, we model YFP expression from our synthetic gene circuit as the convolution of the state specific transcription rates and the states enumerated in *P*_*bound*_(*N*_*TF*_). For the state in which RNAP is solely bound, we give a rate of expression as r (a course-grained parameter representing the basal rate of YFP production). For the TF-RNAP co-bound state, we assign a scaling factor α that represents the change in transcription rate when the TF is bound (acceleration):

(Equation 3) $$YFP_{expression}=r\left(\frac{\left(N_{p}/N_{NS}\right)e^{-\left(\Delta \varepsilon_{p}\right)}}{Z}+\frac{\alpha \left(N_{p}N_{TF}/N_{NS}^{2}\right)e^{-\left(\Delta \varepsilon_{p}+\Delta \varepsilon_{TF}+\Delta \varepsilon_{l}\right)}}{Z}\right)$$

In practice, what we seek to model is the fold change in gene expression, which is the change in expression level relative to the unregulated gene. Based on the partition function and the state specific transcription rates, the fold change in expression then assumes the following form:

(Equation 4) $$fold-change=\frac{YFP_{expression}\left(N_{TF}\neq 0\right)}{YFP_{expression}\left(N_{TF}=0\right)}=\frac{1+\alpha \left(N_{TF}/N_{NS}\right)e^{-\left(\Delta \varepsilon_{TF}+\Delta \varepsilon_{l}\right)}}{1+\frac{\left(\left(N_{TF}/N_{NS}\right)e^{-\left(\Delta \varepsilon_{TF}\right)}\right)\left(1+\left(N_{p}/N_{NS}\right)e^{-\left(\Delta \varepsilon_{p}+\Delta \varepsilon_{l}\right)}\right)}{\left(1+\left(N_{p}/N_{NS}\right)e^{-\left(\Delta \varepsilon_{p}\right)}\right)}}.$$

We then define the following terms $\beta =e^{-\Delta \varepsilon_{l}}$ and $P=\left(N_{p}/N_{NS}\right)e^{-\Delta \varepsilon_{p}}$. As we have measured the value of P in our synthetic circuit to to be 6.6×10^−2^ (Brewster et al., 2012), we safely assume the weak promoter limit simplifies the expression $\frac{1+P\beta }{1+P}\sim 1+P\beta$. The fold change is then written as:

(Equation 5) $$fold-change=\frac{1+\left(N_{TF}/N_{NS}\right)e^{-\left(\Delta \varepsilon_{TF}\right)}\alpha \beta }{1+\left(N_{TF}/N_{NS}\right)e^{-\left(\Delta \varepsilon_{TF}\right)}(1+P\beta )}.$$

We now define the final term in our derivation: $\chi =\left(e^{-\Delta \varepsilon_{TF}}/N_{NS}\right)(1+P\beta )$ which represents the effective component that modifies the TF copy number with the product *χN*_*TF*_ acting in our model as the effective TF concentration. We now re-write the fold change in terms of the effective TF number (*χN*_*TF*_), and the maximal fold $\left(FC_{max}=\frac{\alpha \beta }{1+P\beta }\right)$ as presented in Figure 1 and the main text.

(Equation 6) $$fold-\text{change}=\frac{1+FC_{max}\chi N_{TF}}{1+\chi N_{TF}}.$$

#### Choice of the core-promoter sequence used in the synthetic circuit

Previously we have found that the weak promoter approximation describes *in vivo* measurements of repression of the *lac*UV5 promoter by LacI (Garcia and Phillips, 2011; Brewster et al., 2014). For this study, where we expect to find both activation and repression, we decided to use a weaker promoter for the target gene. This promoter was designed such that $e^{-\Delta \varepsilon_{p}}$ is roughly 1*k*_*B*_*T* lower than that of *lac*UV5 (Brewster et al., 2012). We have confirmed that the basal expression of this promoter decreases as expected and that regulation follows the same quantitative response to LacI as for *lac*UV5 (Brewster et al., 2012). Given that we have previously measured the core-promoter strength used in our synthetic circuit to be at *P* = 6.6×10^−2^ (Brewster et al., 2012), we expect that the approximation 1 + *P* ≈ 1 used in deriving the thermodynamic predictions for the fold change in gene regulation is justified in our work. The choice of the promoter sequence comes with its trade-offs: a weaker promoter sequence, while allowing for a larger window of measurement for activation, potentially limits the ability to measure smaller β values for activation (the weaker the promoter, the larger the range of beta that is constrained to measurable dependence with α - note the weak stabilization limit discussed in the Main Text). Taking all points into consideration, we feel that our choice of the promoter sequence adequately balances competing objectives of detecting activation, and inferring the contributions of α and β, and allowing for strong enough expression to measure 100- to 1,000-fold repression at +1.

#### Concentration manifold derivation

As our primary motivation in this study rests on changing the binding location to explore the regulatory properties of a particular TF, we looked for a way to reformulate the thermodynamic model in such a way as to remove the binding affinity parameter from our consideration (under the assumption that the binding affinity is set primarily by the TF binding sequence which is invariant). This would allow us to infer the intrinsic regulatory features for a TF (the acceleration and stabilization parameters). To do this, consider the following reformulation of the thermodynamic model where the TF concentration is written as a function of the fold change (abbreviated FC below) for a given binding regulatory location (designated by the superscript x or y):

(Equation 7) $$N_{TF}=\left(\frac{1}{\varepsilon^{\left(y\right)}K^{\left(y\right)}}\right)\frac{FC^{\left(y\right)}-1}{FC_{max}^{\left(y\right)}-FC^{\left(y\right)}}=\left(\frac{1}{\varepsilon^{\left(x\right)}K^{\left(x\right)}}\right)\frac{FC^{\left(x\right)}-1}{FC_{max}^{\left(x\right)}-FC^{\left(x\right)}}.$$

Given that we measure TF abundance, we can essentially couple the fold change in regulation between two different positions by allowing the TF concentration to trace out a manifold that specifies the fold change at positions y as a function of the fold change at position x.

(Equation 8) $$FC^{\left(y\right)}=\frac{1+FC_{max}^{\left(y\right)}\left(\frac{\varepsilon^{\left(y\right)}K^{\left(y\right)}}{\varepsilon^{\left(x\right)}K^{\left(x\right)}}\right)\left(\frac{FC^{x}-1}{FC_{\max}^{\left(x\right)}-FC^{\left(x\right)}}\right)}{1+\left(\frac{\varepsilon^{\left(y\right)}K^{\left(y\right)}}{\varepsilon^{\left(x\right)}K^{\left(x\right)}}\right)\left(\frac{FC^{\left(x\right)}-1}{FC_{\max}^{\left(x\right)}-FC^{\left(x\right)}}\right)}.$$

As in the first section of the Methods, we define $P=\left(N_{p}/N_{NS}\right)e^{-\Delta \varepsilon_{p}}$ and $FC_{max}^{p}=\frac{\alpha^{p}\beta^{p}}{1+P\beta^{p}}$ where the superscript *p* represents the regulatory position. We also introduce two new terms for compactness: $\varepsilon =e^{-\Delta \varepsilon_{TF}}$ and *K*^*p*^ = 1 + *Pβ*^*p*^. Assuming the binding affinity is constant between the two locations (*ε* = *ε*^*x*^ = *ε*^*y*^) we achieve a reduction in the manifold:

(Equation 9) $$FC^{\left(y\right)}=\frac{1+FC_{max}^{y}\left(\frac{K^{\left(y\right)}}{K^{\left(x\right)}}\right)\left(\frac{FC^{x}-1}{FC_{max}^{x}-FC^{x}}\right)}{1+\left(\frac{K^{\left(y\right)}}{K^{\left(x\right)}}\right)\left(\frac{FC^{x}-1}{FC_{max}^{x}-FC^{x}}\right)}.$$

Based on this reformulation, the key parameters to consider are the acceleration parameters (couched in the *FC*_*max*_ term as described in Figure 1) and the stabilization parameters at positions x and y (a total of 4 parameters). In a sense, the “concentration manifold” allows us to remove what we consider to be the extrinsic feature of TF regulation (the binding affinity and TF copy number) from the intrinsic features of the TF regulatory response (the regulatory activity as of the TF on RNAP through stabilization and acceleration). We can further simplify the model by taking into account a judicious binding location for the position x. Taking the +1 binding location, where the assumption of steric hindrance in our thermodynamic model for all the TFs surveyed in this work is justified, we set *β*^*x*^ ~ 0 (which makes *K* = 1) and $FC_{max}^{x}=0$. This leads us to the final form of the concentration manifold for a given regulatory position (as a function of the fold change at the +1 position - *FC*^+1^):

(Equation 10) $$FC^{\left(y\right)}=\frac{1+FC_{max}^{y}\left(1+P\beta^{\left(y\right)}\right)\left(\frac{1-FC^{\left(+1\right)}}{FC^{\left(+1\right)}}\right)}{1+\left(1+P\beta^{\left(y\right)}\right)\left(\frac{1-FC^{\left(+1\right)}}{FC^{\left(+1\right)}}\right)}.$$

Now we see that the only parameters that remain in the model are the acceleration and stabilization at position y (the regulatory position under consideration).

#### Culture conditions and data acquisition procedures for microscopy and flow cytometry data

The position dependent regulatory profiles for the 6 TFs -AcrR, AgaR, ArsR, AscG, BetI, CpxR - evaluated at +1 and −61 positions were measured using fluorescence microscopy. At every microscopy session, the TF titration strains (harboring the integrated TF-mCherry fusions in the *ybcN* locus and the synthetic circuit in the *galK* locus) were cultured with companion strains. These include the TF-mCherry fusions lacking the *galK* synthetic circuit integration (necessary to derive the calibration factor to convert the arbitrary fluorescence signal into TF copy number) and TF-mCherry fusion strains with the TF binding site missing from the integrated synthetic circuit (necessary to account for TF titration effect on gene expression). Furthermore, constitutive strains lacking the TF-mCherry fusion (integration in the *ybcN* locus) expressing the integrated synthetic circuit were necessary to compute the fold change in gene expression.

Single colonies of bacterial cultures from freshly streaked LB-Agar plates with appropriate antibiotics are grown overnight in 1 mL of LB in a 37° C incubator shaking at 250 rpm. Cultures are diluted 10^4^- to 10^5^-fold into 1 mL of fresh M9 minimal media supplemented with 0.5 percent of glucose at different aTc concentrations (0, 0.25, 0.5, 1, 1.5, 2, 3, 4, 5, 8, and 10 ng/mL) and allowed to grow at 37° C until they reach an OD600 of 0.1 to 0.2 and harvested for microscopy. 1 μL of cells is spotted on a 2 percent low melting agarose pad (Invitrogen 16520050) made with 1X PBS. An automated fluorescent microscope (Nikon TI-E) with a heating chamber set at 37°C is used to record multiple fields per sample (between 6–12 unique fields of view) resulting in roughly 100 to 500 individual cells per sample.

The calibration factor for the conversion of mCherry fluorescence to TF copy number is quantified by measuring the fluctuations in fluorescence partitioning during cell division (Brewster et al., 2014). Briefly, cells expressing the TF-mCherry fusion protein are grown as described above, and just before imaging 100 μL of cells from different aTc concentrations are pooled together and washed twice with M9-glucose minimal media containing no aTc. Cells are then spotted on 2% low melting agarose pad made with M9-glucose minimal media. Phase images are captured for roughly 150 to 200 fields and their positions are saved for later. These phase images (named as Lineage tracker) will serve as a source file for lineage tracking of the mother-daughter pair. After one doubling time (roughly 1 hour or depending on the doubling time for different TF strains), the microscope stage was returned to the same field of view using the saved position matrix and are imaged again (and named as daughter finder) using both phase and mCherry channels

To measure the regulatory profiles for the CpxR TF position sweep constructs, we used flow cytometry for rapid and reproducible data acquisition. The CpxR-titration strains harboring the position regulation plasmids (See Strains) were cultured in LB + Kanamycin media from single colony inoculates until saturation. A 1:10,000 dilution for each strain was then made in M9 minimal media supplemented with glucose along with the appropriate amount of aTc to titrate CpxR-mCherry levels. We found that the following aTc concentrations 0, 0.5, 1, 1.5, 3, 4, 6 and 8 ng/ml provided a good dynamic range of TF expression while maintaining viability of the CpxR strains. The aTc dilution solutions were made from a stock solution of 1 mg/mL suspended in ethanol and were made fresh prior to the application of the aTc for the M9 culture. After M9 dilution, the strains were grown in 96 well plates to steady state (OD600 of 0.1 – 0.2). Similar to microscopy acquisition procedure, we had constitutive (CpxR-KO) strains transformed with the binding position plasmids along with the CpxR-titration strain transformed with the plasmid having the TF binding sequence removed from the promoter to account for physiological effects of CpxR-mCherry titration to calculate the Fold-change. Cells were diluted between 1:2 to 1:3 fold in PBS media in a 96 well cytometer plate prior to data acquisition and cytometry was performed on a MacsQuant VYB. At the beginning of each run, an initial gating strategy involving the Forward Scatter and Side Scatter area information was used to eliminate background events and samples were run to achieve ~ 60,000 gated events for each position strain at a given aTc concentration.

#### Engineering the titratable TF-mCherry fusion strains

All strains used in this study are constructed from the parent strain *E. coli* MG1655. The TFs investigated in this study include AcrR, AgaR, ArsR, AscG, BetI, and CpxR. Each TF gene is deleted from its wild-type locus and expressed from the *ybcN* locus under the regulation of the *P*_*tet*_ promoter. The autofluorescence strain for each experiment is *E. coli* MG1655 with the corresponding TF knocked out from the wild-type locus. We used the KEIO library (Baba et al., 2006) as the starting point for the construction of the 6 TFs with the titratable TF-mCherry fusion cassette. We selected the corresponding clone from the KEIO library with the TF-knockout (the coding and upstream regions of the TF gene are replaced by a constitutive promoter expressing Kanamycin), and deletion of TF gene from the wild-type locus was performed by P1 transduction of the corresponding knockout from the KEIO collection to the MG1655 *E. coli* strain. The kanamycin cassette was cured using the *frt* flippase expressed from *pCP20* plasmid. Primers listed in Table S1 were then used to amplify the coding sequence (without the stop codon) of the 6 TFs profiled from the MG1655 genome. The amplified coding regions had overhangs for the pTet-AEK-mcherry plasmid that contains the *P*_*Tet*_ promoter in frame with the flexible AEK linker sequence (GCAGAAGCAGCAGCAAAGGAAGCAGCAGCAAAGGCA) and mCherry, and were subsequently cloned into the pasmid via Gibson Assembly to make the respective pTet-TF-AEK-mCherry plasmids. The pTet-TF-AEK-mCherry fusion cassette were then amplified with *ybcN* integration primers for chromosomal insertion using lambda red recombineering assisted by plasmid *pKM208* as described previously (Murphy and Campellone, 2003). We sequenced the regions surrounding the *P*_*Tet*_ promoter and the TF-Linker-mCherry cassette to confirm the regions were free of any mutations. For the final step, the *ybcN* locus was moved using P1 transduction to the TF knockout strain harboring a constitutively expressed TetR integrated at the *gspI* locus. These strains (Table S2) allow for inducible control of TF copy number by titrating TetR repression with aTC (anhydrous tetracycline) and were used for all synthetic circuit measurements presented in the Main Text.

#### Cloning the TF specific binding location gene circuits

The upstream promoter sequence in our synthetic gene circuit was derived from the *P*_*DL5*_ plasmid that contains a modified version of the *lacUV5* promoter sequence as used previously (Brewster et al., 2014). Binding sequences for AcrR, AgaR, ArsR, AscG, BetI, CpxR TFs listed in Table S3 were cloned at the +1 and the −61 locations (relative to the Transcription Start Site) on the plasmid using Gibson Assembly with primers having the TF binding sequences as 5′ overhangs to the priming sequences of the *P*_*DL5*_ vector at the respective binding locations. Primers harboring 40bp homology to the *galK* locus were then used to amplify the promoter region of the plasmid for intergration into the locus using the *pKM208* recombination *E. coli* strains (Murphy and Campellone, 2003). The strains were then sequenced and verified to contain the appropriate regulatory and promoter elements before using P1 transduction to transfer the *galK* locus into the appropriate TF titration strains to make the TF inducible, synthetic circuit strains listed in Table S4.

To clone the synthetic target promoters for profiling the regulatory activity CpxR at multiple binding locations, we designed an approach to make fast and efficient cloning of any TF binding sequence at defined locations ranging from +1 to −112bp relative to the TSS on the unregulated DNA circuit (*P*_*DL5*_). We designed forward and reverse primers to amplify the *P*_*DL5*_ plasmid at defined locations in the promoter sequence (see Table S5). These primers were used to insert the *ccdb* cassette at the precise location upstream of the gene circuit and had overhangs for the typeIIs BbsI restriction site. This allowed for excision of the *ccdB* cassette and cloning of BbsI digested TF binding sequences that had complementary overhangs to the excised region. The *P*_*DL5*_ − *ccdB* plasmids (Table S6) were assembled and transformed into the *Escherichia coli* DB3.1 strain that harbors key mutations in DNA gyrase that tolerates the *ccdB* toxin (Bernard, 1996) for stock curation and sequencing. The plasmids were then incubated with double stranded oligos that had the CpxR *ppiA* binding sequence flanked with the Bbs1 restriction sites and the 4 bp complementary sequence to the digested plasmid. Digestion and ligation of the *ppiA* binding sequence to the *P*_*DL5*_ − *ccdB* position plasmids were done in a single incubation step. The cloned *P*_*DL5*_ − *ppiA* plasmids in Table S7 were then sequenced and transformed into the CpxR titration strain.

### QUANTIFICATION AND STATISTICAL ANALYSIS

#### Data analysis and statistics

Information pertaining to the data plotted in Figures 2, 3, 4, 5, and 6 including the number of replicates and meaning of the error bars can be found in the captions of the respective figures.

#### Data processing steps for microscopy and cytometry data

To process the regulatory data for the 6 TFs profiled at +1 and −61, we took the microscopy images and segmented individual cells using a modified version of the MATLAB code Schnitzcells (Rosenfeld et al., 2007).We use this code to segment the phase images of each sample to identify single cells. Mean pixel intensities of YFP and mCherry signals are extracted from the segmented phase mask for each cell. The autofluorescence is calculated by averaging the mean intensity of the autofluorescence strain in both mCherry and yfp channels and is subtracted from each measured YFP or mCherry value. Total fluorescence for each channel is obtained by multiplying the mean pixel-intensity with the area of the cell. Fold change in expression for a given binding site is calculated by the ratio of total fluorescence of strains expressing the TF to the strains with no TF. For partitioning statistics to estimate the calibration factor, mother-daughter pairs are first automatically identified and verified manually to ensure cells made exactly one division. The mean pixel intensity and area of the mother-daughter pairs are obtained. The background fluorescence is estimated as described previously (Ali et al., 2020) using the inverse mask of individual frames. The sum and difference in fluorescence of the two daughters were then used to find the conversion factor, *v*, between fluorescence and number of TFs using the equation (*I*_1_ − *I*_2_)^2^ = *v*(*I*_1_ + *I*_2_), which stems from the assumption of binomial partitioning of TFs at cell division (Rosenfeld et al., 2007).

For the CpxR position cytometry data, we adapted a robust data analysis procedure (Razo-Mejia et al., 2018) to computationally gate events to ensure reproducible Fold-change measurements for a given position across replicates. For a given position strain replicate measurement, we collected the data across all the aTC concentrations and proportionally binned the single flow cytometry events into 16 RFP intervals (intervals were off unequal size in RFP space with the number of cells in each bin more or less constant). We then took these binned events and gated them using the *log*_10_ values of the Forward Scatter and Side Scatter area profiles for each event (referred to as FSC and SSC respectively) to improve the likelihood that the final retained events were single cell measurements. To construct this gate, we computed the mean and covariance matrix for each dataset for every RFP bin and used these statistics to fit an ellipsoid to the full dataset according to the following formula:

(Equation 11) $${\left[\begin{array}{l} FSC\\ SSC \end{array}\right]}^{T}\Sigma^{-\frac{1}{2}}\left[\begin{array}{c} FSC\\ SSC \end{array}\right]\leq \alpha ,$$

(Equation 12) $$with\Sigma =\left[\begin{array}{ll} Var(FSC) & Cov(SSC,FSC)\\ Cov(FSC,SSC) & Var(SSC) \end{array}\right].$$

This step retains events that are within a particular distance from the center of the ellipsoid using an appropriate value for the cut-off (alpha). We based the value of the cut-off on the following rationale: As each event is essentially a vector of *log*_10_ values for the FSC (Forward Scatter) and SSC (Side Scatter), we assume the joint values are normally distributed, which translates to a distance metric that is a chi-square random variable (the sum of two normally distributed entities is chi-square with 2 degrees of freedom). We selected α as the 5^*th*^ percentile of values from the cumulative distribution, and events within the cutoff were taken to be single cell measurements used to compute the fold change values presented in the results section. The resulting gated events for each of the 16 RFP intervals were pooled from all position strains and replicates, and we excluded events with RFP measurements below a certain threshold determined by visually assessing the the fold change profile of the “control” (*DelBS*) circuit. The YFP signal for these events had large fluctuations and we reasoned that the flow-cytometry approach probably fails in measuring cells at lower TF copy number. The retained events were then binned proportionally into 22 intervals, and the median RFP and Fold change values for each interval was reported as the representative measurements for that bin. The choice of intervals at this step (with the exception for very large bin sizes) does not seem to appreciably alter the main findings from our inference into the acceleration and stabilization for the CpxR binding locations (see Figures S5 and S6 for details).

#### Measurement of TF abundance in flow cytometry experiments: Converting the mCherry signal to TF copy number

To measure the regulatory response of the CpxR regulated promoter at 22 binding locations, we used flow-cytometry to measure TF abundance and target gene expression for cells at steady state. Our goal was to look at the regulatory level of the target gene as a function of TF copy number (Brewster et al., 2014), which required TF abundance measurements to be converted from arbitrary fluorescent units to copy number of the TF-mCherry fusion molecules in the cell. To convert the arbitrary mCherry signal from the flow cytometer to TF copy number, we took the flow cytometer measurements of the Fold change response curve as a function of mCherry levels at the +1 position and compared it to the measurements we made using microscopy (see Figure S1). As we show in the Main Text this regulation is consistent with steric hindrance (*FC*_*max*_ = 0, *β* = 0 (Bintu et al., 2005)). We then extracted the parameter, χ, from both the microscopy and cytometry curves using the thermodynamic model we present in the Main text and compared them. Details on this approach can be found in the STAR Methods (Quantification and statistical analysis) in the subsections Parameter fitting and inference for position dependent fold change regulation data and Using the concentration manifold parameters to generate the thermodynamic model in FC versus *N*_*TF*_space.

We reasoned that the while the value inferred for the cytometry curve (*χ*_*RFP*_) and microscopy curve (χ) are different, the relation *χ*_*RFP*_*RFP* = *χN*_*TF*_ will be true according to the thermodynamic model of simple repression:

(Equation 13) $$fold-change_{+1}=\frac{1}{1+\chi_{RFP}RFP}=\frac{1}{1+\chi N_{TF}}\text{.}\;$$

This leads to the following interpretation of the quantity *χ*_*RFP*_*RFP*:

(Equation 14) $$\chi_{RFP}RFP=\lambda_{RFP}\frac{1+P\beta }{1+P}RFP=\lambda_{\text{mic}\;}\frac{1+P\beta }{1+P}\mu RFP=\chi N_{\text{TF}\;}.$$

Here the parameter λ represents the binding affinity of CpxR with the subscript denoting the units it was measured in. In Figure S1, we show the regulatory curves from the +1 parallel measurements for the cytometry (magenta curve and data points) and microscopy (black curve). The difference between the curves is expected as the units of χ (*χ*_*RFP*_ for cytometry and *χ*_*mic*_ for microscopy) are different, and the scaling factor μ (in units of TF per RFP signal) is required to make the curves the same. We find *χ*_*RFP*_ = 2.33×10^−4^ (magenta curve) and χ = 1.3×10^−4^ with the value of *μ* = 1.8. We used this value of μ and multiplied the RFP signal in the cytometry data to scale the x axis in Figures 5C and 6E.

#### Parameter fitting and Inference for position dependent fold change regulation data

We interpret the promoter regulatory data from the 6 TFs surveyed at the +1 and −61 binding locations through the thermodynamic model as specified in the results section. The fold change data for the TF-position strain (See Data processing steps for microscopy and cytomtery data section for details) as a function of *N*_*TF*_ was fit to Equation 6 with the aim of extracting the best-fit value of the *FC*_*max*_ and χ (the product of the stabilization effect and the TF binding affinity). We used a bootstrapping procedure to generate confidence intervals for the both the *FC*_*max*_ and binding affinity parameters, and for each of the TFs surveyed we fit the +1 and −61 parameter sets independently. The bootstrapping procedure resampled the data points from the fold change versus RFP curve for a given TF-binding location across all replicate datasets 1000 times. For each iteration, fold change replicate data points from a given induction conduction were sampled to generate a possible regulatory response as a function of TF-copy number. Each of these resampled curves were then fit to the thermodynamic model outlined in Figure 1A using a non-linear least-squares fitting procedure to determine the optimal fit for the values of *FC*_*max*_ and χ parameters. As seen in Figures 4C and Figures 4D, we report the means and confidence intervals for these two parameters and plot the curve generated by taking the expected value of the thermodynamic model conditioned on the model parameters along with the 95% confidence interval.

For the CpxR position sweep data, we used the concentration manifold formalism to delineate the values of the acceleration and stabilization parameters. For positions that showed discernible regulation (12 out of the 22 upstream positions profiled), we assume that the binding affinity is constant between the regulatory positions as the only changing variable is the binding location (the binding sequence is the same) and recast the binned data from the Fold-change versus RFP replicates using the concentration manifold formalism as detailed in the STAR Methods (see Concentration Manifold Derivation). To sample the probability space of the acceleration and stabilization parameters for a given binding location, we started by inferring the joint posterior distribution *FC*_*max*_ and *K* = 1 + *Pβ* using a Bayesian approach that relates the parameters underlying our thermodynamic model to the data according to the following relation:

(Equation 15) $$p\left(FC_{\max},K|FC\right)=\frac{L\left(FC|FC_{\max},K\right)p\left(FC_{\max},K\right)}{p(FC)},$$

where the term on the left hand side is the posterior distribution of the parameters (*FC*_*max*_ and *K*). The terms on the right hand side represent the likelihood of the data given the parameters(*L*(*FC*|*FC*_*max*_,*K*), the prior distribution of the parameters (*p*(*FC*_*max*_,*K*)), and lastly the distribution of the data itself *p*(*FC*). Each of the 12 regulatory positions were fit separately using the Bayesian inference procedure, we specified our likelihood function as a normal distribution of the form:

(Equation 16) $$L^{K}\left(FC^{k}|FC_{max}^{k},K^{k}\right)=\prod_{i}^{\text{DataPoints}\;}Normal\left(FC_{i}^{k}|u=FC_{prop\;}^{k}\left(FC_{i}^{+1};FC_{max}^{k},K^{k}\right),sd=\theta^{k}\right).$$

The superscript k represents an upstream binding location for the CpxR TF and the subscript i represents the data points for a given position regulatory dataset. The proposed Fold-change value *FC*_*prop*_ from the model takes the form:

(Equation 17) $$FC_{prop\;}\left(FC_{i}^{+1};FC_{max},K\right)=\frac{1+FC_{max}K\left(\frac{1-FC_{i}^{+1}}{FC_{i}^{+1}}\right)}{1+K\left(\frac{1-FC^{+1}}{FC_{i}^{+1}}\right)}.$$

The crux of the Bayesian approach to model inference is to simulate candidate draws of the joint posterior distribution of the *FC*_*max*_ and *K* parameters by proposing candidate values from the prior distribution, generating the thermodynamic curve, and evaluating the likelihood function. A transition in the jointly sampled parameter space from one set of parameter values to another is based on the premise that parameter sets will be sampled in proportion to the probability of the posterior distribution (as long as the sampling chain is drawing from the stationary distribution). This process is repeated until a given number of draws have been made from the joint posterior distribution. The results of this inference procedure are used to draw the model curves in Figure 5C and we use the relation between the sampled parameters (*FC*_*max*_ and *K*) and the acceleration and stabilization parameters as detailed previously in the methods section. The sampling procedure was implemented with the PyMC3 package that utilizes the NUTS sampler, a particular implementation of the Hamiltonian Monte-Carlo algorithm, to sample the joint posterior distribution (Hoffman and Gelman, 2014).

We used a uniform distribution as the priors for both the *FC*_*max*_ and *K* model parameters with appropriate bounds for each parameter. For *K*, we ensured that the lowest potential value is 1, in line with the assumptions from the derivation of the concentration manifold formalism. We checked the inferences from each position to ensure that the bounds we enforced on both parameters were appropriate and that the sampled values were not tending toward the edge of the sample space. Furthermore, we cast the σ parameter (the standard deviation) of the position specific likelihood function as a hyper-parameter in our sampling procedure and set the prior distribution as uniform over a defined interval with the lower bound = 0. Overall, our inference approach allowed us to ensure precise inference of α and β and for 11 out of the 12 regulatory positions (See Table 2).

To get the stabilization values from this inference procedure, we simply used the following relation between the *K* and β and the fact that the polymerase occupancy has a measured value of 6.65×10^−2^ in our synthetic promoter,

(Equation 18) $$\beta =\frac{K-1}{P}.$$

Given the stabilization value for a draw in the chain, we then find the corresponding acceleration value using the jointly sampled *FC*_*max*_ value and the relation:

(Equation 19) $$\alpha =\frac{FC_{max}}{\beta }\left(1+\left(\frac{P}{1+P}\right)(\beta -1)\right).$$

Table 2 lists the inferred acceleration and stabilization parameters. We report the median values of the inference chain along with the bounds that encompass the 68%th percent Bayesian credible interval of the parameters. For position −74, the inference estimates are not precise due to the phenomenon of the “weak stabilization” limit as discussed in the Main text. Examining the posterior distribution of the *FC*_*max*_ and *K* parameters, we find the values of the 68% credible interval for this positions (both α and β) to encompass both coherent and incoherent regimes.

#### Inference plots for CpxR position sweep data - posterior distributions for the *FC*_*max*_ and K

As detailed in the Methods section, we used a particular formulation of the thermodynamic model (Forcier et al., 2018) that used the TF abundance measurements from cytometry to rewrite the fold change data for a given regulatory position as a function of the fold change at the +1 position (see the STAR Methodssection on the Concentration Manifold Derivative). The benefit of this re-formulation was the ability to remove the binding affinity parameter and find the “intrinsic” regulatory parameters of the TF (α and β).

To extract α and β for a given regulatory position, we began by inferring the *FC*_*max*_ and the *K* = 1 + *Pβ* parameters using a Bayesian Sampling approach ((Hoffman and Gelman, 2014)) as detailed in the inference procedure (see STAR Methods Section for details). Figure S2 shows the result of the inference of the posterior distribution for the two parameters for each of the 12 regulatory positions. The first and third column shows the outcome of the Bayesian approach to sampling the posterior distribution for *FC*_*max*_ and *K* parameters, and the second and fourth column presents the resulting transformation into the α and β joint distribution space. The plots are arranged in order from highest β at the top left of the figure to smallest β at the bottom right. As seen, there is tight coupling for the joint distributions between the *FC*_*max*_ and the *K* with an inverse dependence between the two parameters for activation (high values of *FC*_*max*_ are sampled jointly with low values of *K* and vice versa) and opposite for the single repressing position −54 (low values of *FC*_*max*_ are sampled jointly with high values of *K* and vice versa). Arranging them in order of decreasing β serves to demonstrate an important point: the correlation between inference of α and β becomes stronger as β gets smaller (i.e., as *K* approaches 1). In all cases, we can infer *FC*_*max*_ effectively but the individual value of one regulatory parameter depends strongly on the other because when *βP* is small, *FC*_*max*_ ≈ *αβ* and *K* ≈ 1, and thus β and α have a strong inverse relationship. When plotted on log axis this appears as a straight line (of slope −1) and the domain of β from the inference sampling becomes less constrained. For one of the 12 regulatory positions (−74), we are unable to separate α and β with any certainty and the inferred values encompass both incoherent and coherent regulatory outcomes for the appropriate *FC*_*max*_ (Transtrum et al., 2015).

#### Using the concentration manifold parameters to generate the thermodynamic model in fold change versus *N*_*TF*_ space

The parameters inferred using the concentration manifold approach were used to construct the thermodynamic model curves in Figures 5C and 6E. To accomplish this, we used the *K* = 1 + *Pβ* and *FC*_*max*_ values inferred for each of the 12 regulatory positions (see STAR Methods: Parameter fitting and Inference for position dependent fold change regulation data). To transform the Markov chain of *K* values to the χ for the main thermodynamic model (Figure 1, and STAR Methods) requires the binding affinity of the TF (the final remaining term in χ), and we inferred the binding affinity from the datasets encompassing all 13 regulatory positions for the CpxR TF (the 12 upstream regulatory positions and the one immediate downstream positions − the +1 position). The binding affinity was treated as a global parameter in our Bayesian inference scheme and was inferred according to the following model:

(Equation 20) $$\prod_{k}^{Position}\prod_{j}^{Data\mathrm{points}}Normal\left(FC_{j}^{k}|u=FC_{Themodynamic(j)}^{\left(k\right)}(\lambda ),sd=\sigma^{\left(k\right)}\right))$$

with the mean of the likelihood function specified by the thermodynamic model outlined in the STAR Methods section Thermodynamic model for single TF regulation and takes the following form:

(Equation 21) $$FC_{Thermodynamic(j)}^{k}=\frac{1+FC_{max}^{k}\chi^{k}N_{TF(j)}^{k}}{1+\chi^{k}N_{TF(j)}^{k}},$$

where *χ*^*k*^ = *λ*(1/1 + *P*)*K*^*k*^.The parameter λ, is the global parameter representing the scaled binding affinity of the CpxR TF $\left(\lambda =\left(1/N_{NS}\right)e^{-\Delta \varepsilon_{TF}}\right)$ and is assumed to be constant across the regulatory positions assessed in this work. *FC*^*k*^ is the general thermodynamic model specified in Equation 6 with the object of our inference to infer λ. For the 12 upstream regulatory positions, as the chains of *FC*_*max*_ and *K* were available from the inference of the concentration manifold dataset, we inferred the global λ parameter to the datasets for each of those positions with the values of these two parameters determined from the mean of their respective Markov chains. For the +1 dataset, the parameter *FC*_*max*_ was set to 0 as determined in Figure 4A, and the *K* was set to 1 in keeping with the assumption of steric hindrance. The mean value of the Markov chain λ inferred from this model, along with the scaling factor (see SI section - Converting the mCherry signal to TF copy number) was used to generate the thermodynamic model curves (*mean* ± 2*σ*) as seen in Figures 5C and 6E.

#### Robustness of the concentration manifold results

To analyze the fold change data of our experiments we bin the single-cell fluorescence measurements to find the average fold change of cells with similar TF concentrations (mCherry levels). To accomplish this we divided the data into a specified number of bins based on the proportion of the total data points and calculated the ensemble fold change from the cells in each bin. In Figures S5 and S6 we show how the determination of the parameters β and α depends on this choice. In these plots the inferred value of alpha and beta for each of the 12 positions are shown for 14 different bin numbers (6, 8, 10, 12, 14, 16, 18, 20, 24, 26, 28, 30, 32, 36) number of bins and plotted against the value found with 22 bins (used in the main text). The quatity on the y axis is a measurement of the degree to which the values of β (or α) differ from the reference bin across all regulatory positions, and is computed by taking the mean of the *log*_10_ ratios between the reference bin and the bin under consideration for each of the 12 regulatory positions. In Figure S5, we see that the inferred value of β for other bin sizes is tight across most of the regulatory positions with more substantial deviations from 1 as the number of bins becomes very small (<12). This phenomenon is an indication to the degree the larger bin sizes (smaller bin numbers) inadequately convey the curvature inherent in the data, pushing more of the regulatory positions to overstimate the degree of curvature for certain regulatory positions. Figure S6 shows this same measure for the inferred values of α, where we we once again see consistency in the inferred value across most of the regulatory positions except for the small number of bins as seen in Figure S5. Crucially, the inference of α and β is not critically sensitive to the choice of bin size above 12 bins.

#### Testing alternative models of transcriptional regulation for the CpxR position sweep data

In this section, we present an alternate interpretation of the CpxR position sweep data. Specifically we explore if the data can be explained by a simpler model with only one unique regulatory parameter for a given concentration manifold dataset. Specifically, the models we will evaluate in this section will assume the TF operates only through (de)stabilization of RNAP (*α* = 1).

For the model inference runs, we set the energy of the promoter used in our synthetic circuit (DL5 promoter sequence (Brewster et al., 2012)) as a global parameter across the 12 regulatory positions and allowed each position to infer its own stabilization (β) value, with *FC*_*max*_ and the *K* terms as defined in the concentration manifold section:

(Equation 22) $$FC^{y}=\frac{1+FC_{max}^{\left(y\right)}K\left(\frac{1-FC^{\left(+1\right)}}{FC(+1)}\right)}{1+K\left(\frac{1-FC(+1)}{FC^{\left(+1\right)}}\right)},$$

(Equation 23) $$FC_{max}^{\left(y\right)}=\frac{\beta^{\left(y\right)}}{1+\left(\frac{P}{1+P}\right)\left(\beta^{\left(y\right)}-1\right)},$$

24 $$K=1+P\beta^{\left(y\right)},$$

where *FC*^(*y*)^ and $FC_{max}^{\left(y\right)}$ represent the fold change and *FC*_*max*_ effective parameter when the binding site is introduced at position *y* on the promoter. Note that *FC*_*max*_ no longer has the acceleration parameter. For the Bayesian inference scheme, we set the prior of the DL5 promoter sequence energy as a uniform distribution between the values −10 *k*_*B*_*T* and −2 *k*_*B*_*T*. The sequence energetics have been measured in prior work as −6.5 *k*_*B*_*T*, as such we believed this was a reasonable interval for the chain to sample. For the position dependent stabilization parameters, we used a uniform distribution with acceptable bounds for all activation positions. We modeled the likelihood function as product of normal distributions (assuming the global likelihood function is a product of the individual position specific likelihood functions) with the mean of these position specific distributions as the theoretical fold change value generated from the thermodynamic model conditioned on the parameters. To ensure that differences in the fold change profiles between strong activation positions and weak activation positions was adequately conveyed in the likelihood function, we made the standard deviation for the respective position specific normal distributions a hyperparameter in our model, with the final form of the likelihood function as follows:

(Equation 25) $$\prod_{i}^{Position}\prod_{j}^{Data\;Points}Normal\left(FC_{j}^{i}|u=FC_{stabilization\;(j)}^{\left(i\right)}\left(P,\beta^{i}\right),sd=\sigma^{\left(i\right)}\right),$$

and:

(Equation 26) $$FC_{stabilization\;(j)}^{\left(i\right)}=\frac{1+FC_{max}^{\left(i\right)}\left(P,\beta^{\left(i\right)}\right)K\left(P,\beta^{\left(i\right)}\right)\left(\frac{1-FC_{j}^{\left(+1\right)}}{FC_{j}^{\left(+1\right)}}\right)}{1+K\left(P,\beta^{\left(i\right)}\right)\left(\frac{1-FC_{j}^{\left(+1\right)}}{FC_{j}^{\left(+1\right)}}\right)}.$$

We initialized the inference procedure as sampling from a global vector, θ, that contained the global and position specific parameters in our model,

(Equation 27) $$\theta =\left[P^{energy\;},\beta^{-48},\beta^{-50},\beta^{-54}\ldots ,\beta^{-80},\beta^{-82}.\right]$$

The inference procedure was run for 50000 runs initialized on 4 different chains to ensure adequate sampling of the joint parameter space. The fits for the “stabilization only” model are shown in Figure S3 with the values of the 68%th percent Bayesian credible interval reported in Table S8. In this figure, we plot the results of the thermodynamic model from the inference sampling. It is clear that the stabilization only model fails in capturing the highest activation position (−64) and the curvature seen in some positions (−50, −54). This failure to explain strong activation is expected, as in a model without acceleration (*α* = 1) the maximum possible fold change is constrained by the individual occupancy of the promoter by RNAP (the maximal possible fold change is roughly 1/*P* for weak promoters or more precisely (1 + *P*)/*P* if the weak promoter assumption is lifted (Phillips and Milo, 2009)); intuitively, in this model without acceleration if the constitutive promoter has polymerase occupancy 10% of the time, the largest fold change possible is 10 (corresponding to 100% occupancy). To reach the fold change values obtained in the CpxR position sweep data, the inferred value of *P* needs to be at least ~ 10 fold lower than the value of *P* measured in previous work (Brewster et al., 2012). As such, we feel confident that this model can not sufficiently describe our data.

It is worth noting that even if this assumption is incorrect and in reality *P* is much smaller than we expect from previous measurements, the model without acceleration still does not describe the data well; the theory does not match the curvature of the data seen in some positions such as −50, −54, and −60 (Figure S3). This feature highlights the importance of acceleration (α) in explaining these positions and the regulation data at large.

#### Physiological effects of TF titration on synthetic circuit expression

One concern for the gene expression measurements was separating the fundamental regulatory role of a TF from the apparent expression changes due to potential physiological effects such as slowing growth rates from, for instance, high inducer concentrations or changing TF concentration in the cell (Berthoumieux et al., 2013; Klumpp and Hwa, 2014; Keren et al., 2016) brought about by inducing the TFs to different levels. Increasing concentrations of the TF in the cell could potentially alter YFP expression by turning on or off genes involved in global regulation of translation or through a host of post-transcriptional events. In Figure S4, we do not see a major perturbation in synthetic circuit expression for most of the TF titration strains where YFP expression hovers at the FC=1 line as the TF concentration increases.

## Supplementary Material

1

## Figures and Tables

**Table 1.** Channel settings used for the cytometry acquisition

Emission filter	Channel	Voltage

525/50 nm	B1	510 V
615/20 nm	Y2	524 V
561/4 nm	FSC	405 V
561/4 nm	SSC	315 V

All cytometry measurements were done on a MacsQuant VYB with the listed channel settings. A threshold using the forward scatter (FSC) and side scatter (SSC) area measurements was used initially to gate the event data. Subsequent data processing steps to convert these cytometry measurements to the fold change plots in Figures 5 and 6 can be found in the STAR Methods.

**Table 2.** Inferred acceleration and stabilization parameters

Binding position	α (alpha)	β (beta)

−48	$3.506_{2.898}^{4.008}$	$116.257_{66.453}^{150.131}$
−50	$0.675_{0.612}^{0.736}$	$161.766_{108.281}^{199.140}$
−54	$0.049_{0.047}^{0.051}$	$34.496_{23.154}^{43.447}$
−56	$3.38_{1.745}^{4.496}$	$14.723_{5.304}^{21.647}$
−58	$3.107_{1.635}^{4.127}$	$10.519_{3.796}^{15.386}$
−60	$2.161_{1.974}^{2.33}$	$219.757_{151.499}^{270.481}$
−64	$24.911_{14.911}^{31.849}$	$29.545_{12.984}^{44.169}$
−70	$6.995_{3.101}^{9.748}$	$6.224_{1.776}^{9.316}$
−72	$0.813_{0.451}^{1.053}$	$20.905_{7.678}^{30.744}$
−74	$2.329_{0.467}^{3.944}$	$2.982_{4.789\times 10^{-6}}^{4.685}$
−80	$0.289_{0.197}^{0.355}$	$27.025_{10.944}^{37.948}$
−82	$0.271_{0.182}^{0.334}$	$24.811_{9.835}^{35.501}$

Shown are median values of the inference chain along with the bounds that encompass the 68% Bayesian credible interval of the parameters α and β inferred from the data presented in Figure 6C. The “weak stabilization limit” limits the precision estimates for position −74.

**KEY RESOURCES TABLE:** 

REAGENT or RESOURCE	SOURCE	IDENTIFIER

Bacterial and virus strains		

*Escherichia coli,* strain BW135112	KEIO collection	NZ_CP037857

Chemicals, peptides, and recombinant proteins		

M9 minimal media	BDDiagnostics	DF0485–17

Critical commercial assays		

ZR Plasmid Miniprep	Zymogen	Cat#D4015

Experimental models: Organisms/strains		

*Escherichia coli,* strain MG1655 – Complete list of modified *E. coli* strains can be found in Tables S2 and S4	This Paper	CGSC#6300

Oligonucleotides		

Position Sweep Primers for *ccdB* cassette insertion into pZS25LongUPDL5-delbs-YFP (see Table S5 – Supplement Section)	Primer design using custom-made python script, synthesized by Genewiz	N/A
Primers for amplifying the TF gene cassettes from the MG1655 genome – See Table S1	Genewiz	N/A

Recombinant DNA		

pDONR P4-P1r	Thermo Fisher (Invitrogen-Life Technologies)	N/A
*ccdB* Position cloning strains (Table S6)	This Paper	N/A

Software and algorithms		

MATLAB	https://www.mathworks.com/products/matlab.html	N/A
Python	https://www.python.org/downloads/	N/A
PyMC3	https://docs.pymc.io/en/stable/about.html	N/A

## Highlights

- Synthetic biology approach to dissect the fundamental role of TFs
- Model infers the role of two regulatory mechanisms: acceleration and stabilization
- TFs use different degrees of each mechanism based on binding location
