# Supplementary material for: Quantifying the regulatory role of individual transcription factors in Escherichia coli
Source: Cell Rep. Author manuscript; Available in PMC 2021 Dec 13. (PMC8667592; doi:10.1016/j.celrep.2021.109952)
Supplement: 1 [file NIHMS1756263-supplement-1.pdf]

**Cell Reports, Volume *37***

## Supplemental information

# Quantifying the regulatory role of individual transcription factors in *Escherichia coli*

**Sunil Guharajan, Shivani Chhabra, Vinuselvi Parisutham, and Robert C. Brewster**

# Supplemental Figures and Tables

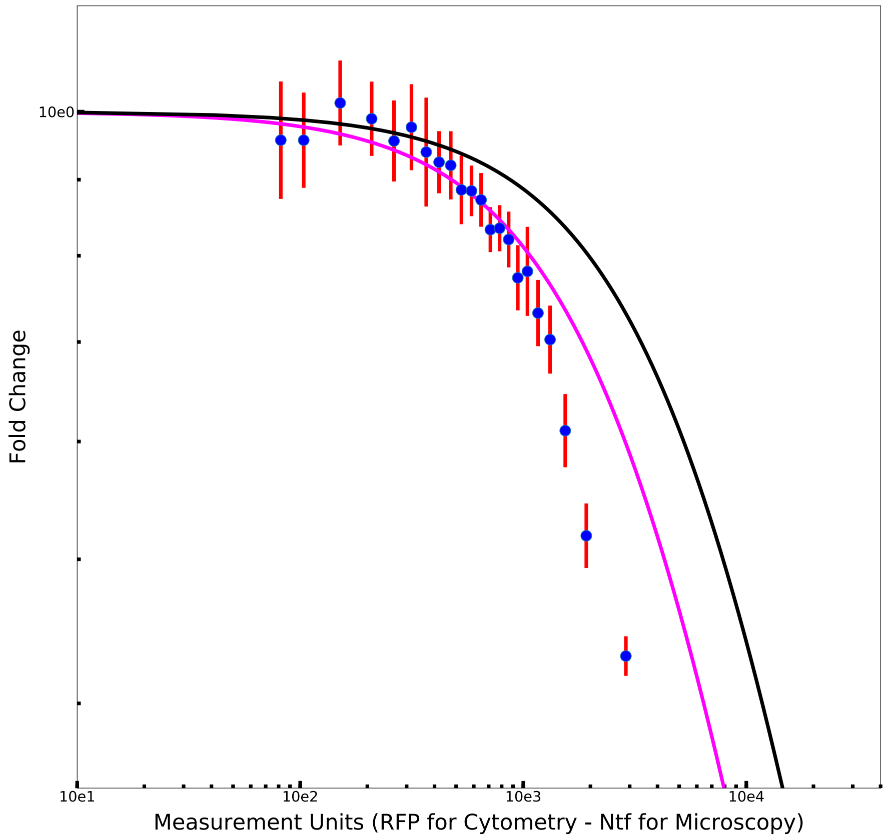


Figure S1: **Plot of the fold-change in regulation for the +1 binding site from the cytometry and microscopy approach.** We used the +1 data measured in parallel for both microscopy and cytometry to generate the scaling factor that would allow for conversion of arbitrary fluoresence in cytometry to TF copy number. The data points represent the measured fold-change values from the cytometry approach and the magenta line represents the fit generated from the thermodynamic model where $\chi_{\mathrm{RFP}}$ is fitted to the global CpxR position regulation dataset (See Methods Section "Using the concentration manifold parameters to generate the thermodynamic model in FC vs $N_{\mathrm{TF}}$ space" for details). Data points (in blue) are the means of 4 replicate measurements ($n = 4$) with the error bars representing the standard error of the mean. The black line represents the model where $\chi$ is fit to the microscopy data. Note that the $x$-axis for the cytometry curve and data points is in RFP units while for the microscopy curve it is in TF copy number. The difference between the curves can be removed by scaling the value of $\chi_{\mathrm{RFP}}$ by $\mu$, and this resulting scaling value ($\mu = 1.8$ TFs/RFP unit) is the factor that converts the RFP measurements to TF copy number.

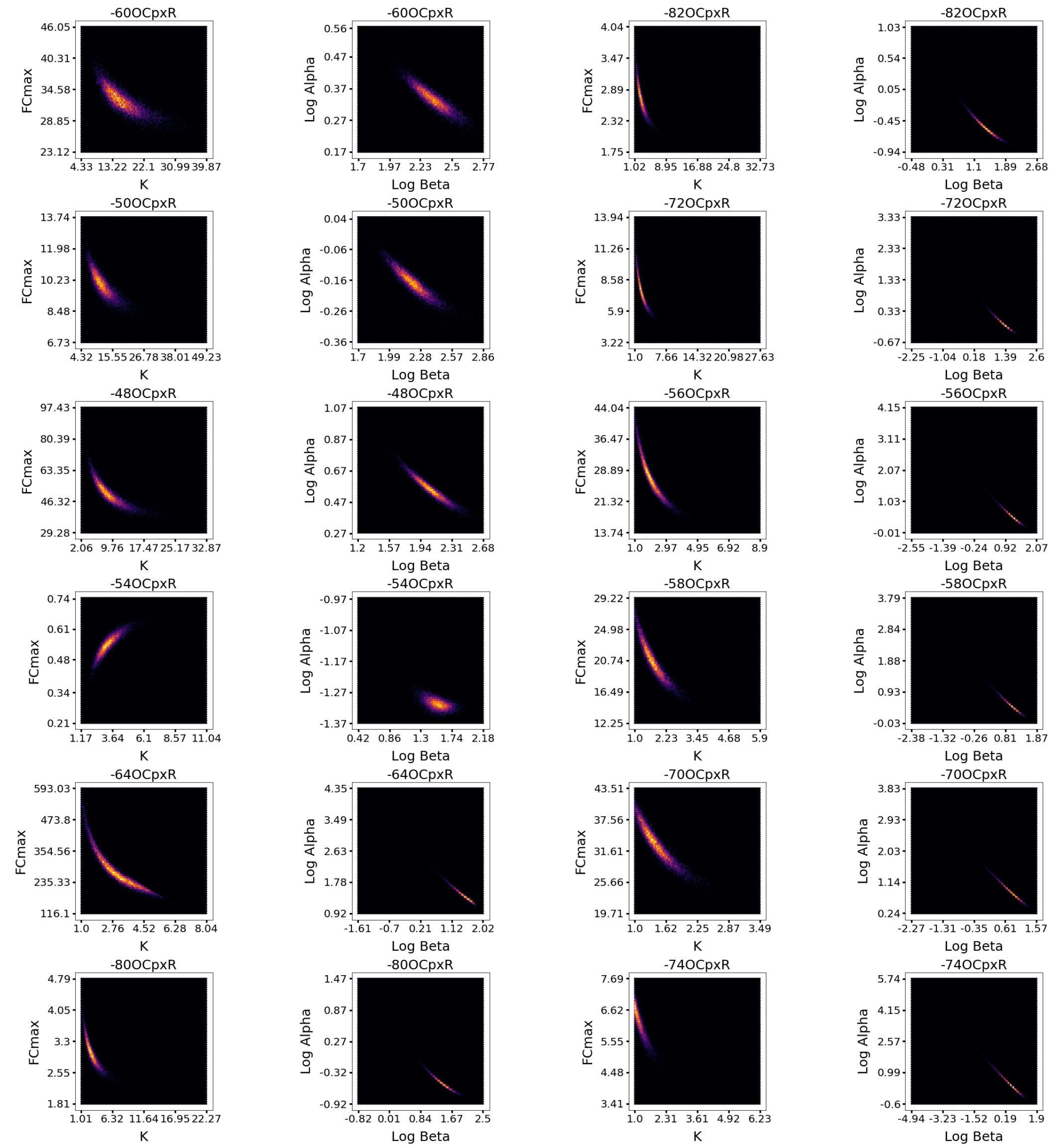


Figure S2: **Inference plots for the Stabilization Only Model.** Plots of the $FC_{max}$ and $K$ from the model inference of the concentration manifold approach in the first column with the second columns representing the transformation into the joint space of $\alpha$ and $\beta$ as presented in the main text. The positions are arranged from highest activation to moderate repression. Note the strong inverse dependence between the $FC_{max}$ and $K$ parameters for the activation positions that is reversed for the repression position $-54$. A log-log plot of the $\alpha$ and $\beta$ parameters will yield a straight line if the $K$ is 1, and the sampled space for $\alpha$ and $\beta$ will be large preventing inference on the mode of regulation for the given regulatory position.

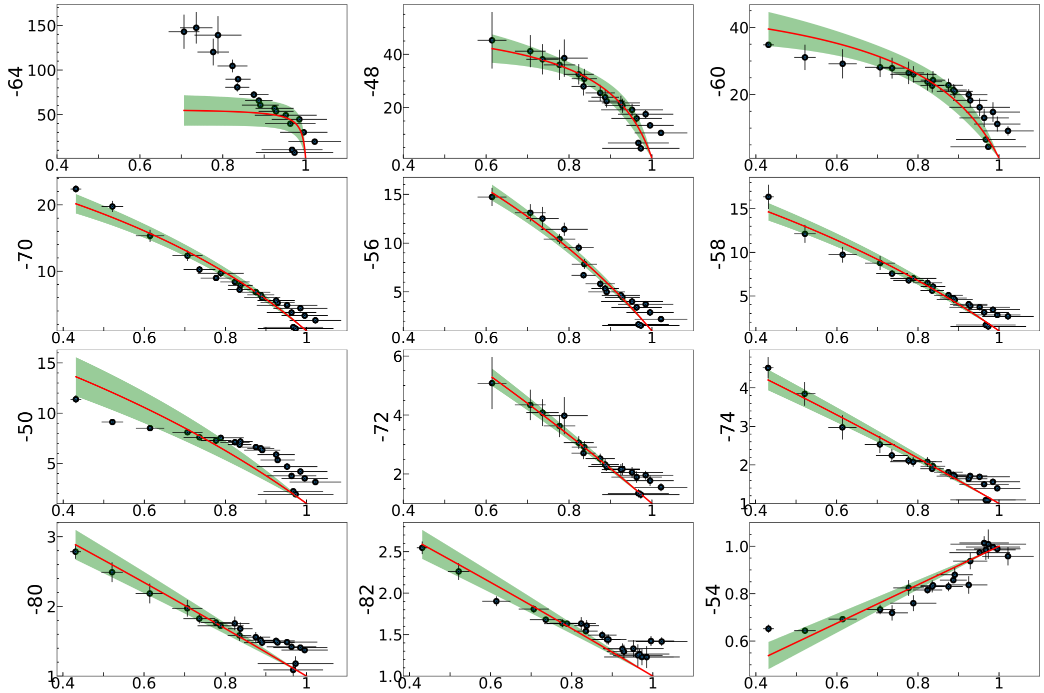


Figure S3: **Plots for the Stabilization Only Model.** The position plots are arranged from highest to lowest activation positions. The regulatory contours are generated from the inferred parameters according to a model that lacks acceleration ($\alpha = 1$). The black data points are from the concentration manifold approach as described in the Main Text (See Figure 6C for details). The red solid line represents the model expectation conditioned on the inferred parameters (the position specific $\beta$ and the global $P$ parameter) with the green shaded area as 2 standard deviations from this expectation. Note that the stabilization model fails to capture the data for positions with strong curvature as well as the highest activation position ($-64$).

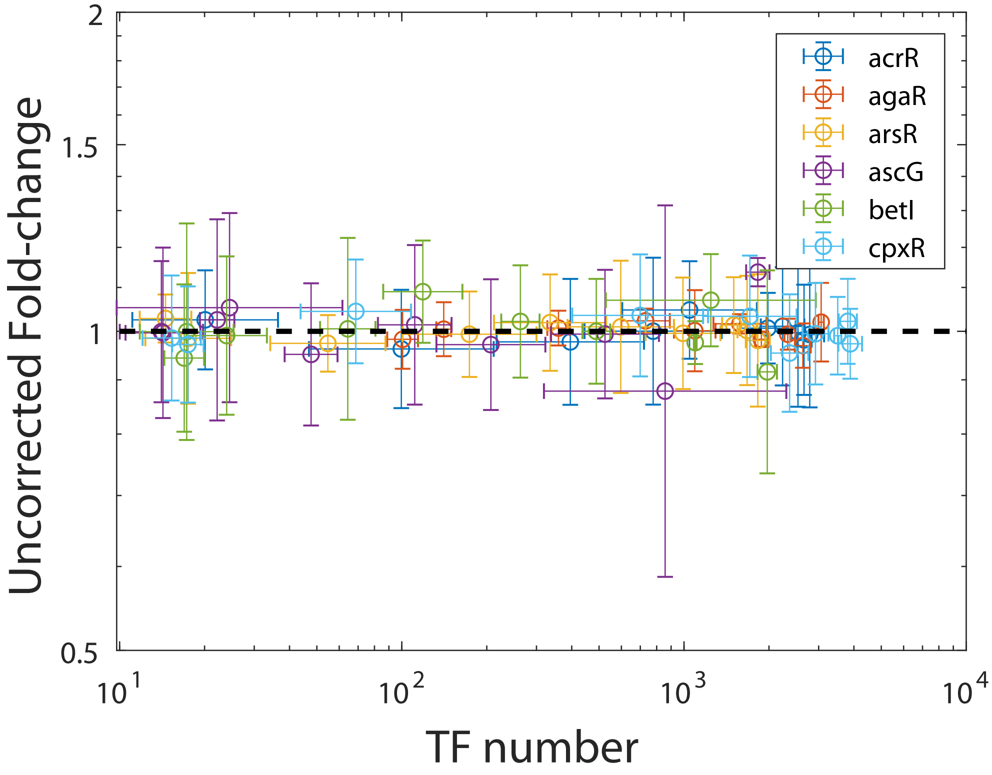


Figure S4: **Physiology effects of TF-titration on YFP expression.** TF-titration strains regulating the control synthetic circuit (*Psz2-DelBs*) as measured using microscopy. The dashed black line represents FC=1, and we see most of the TFs do not appreciably alter YFP expression simply through the titration effect. Data points (in blue) are the means of 3 replicate measurements ($n = 3$) with the error bars representing the standard error of the mean

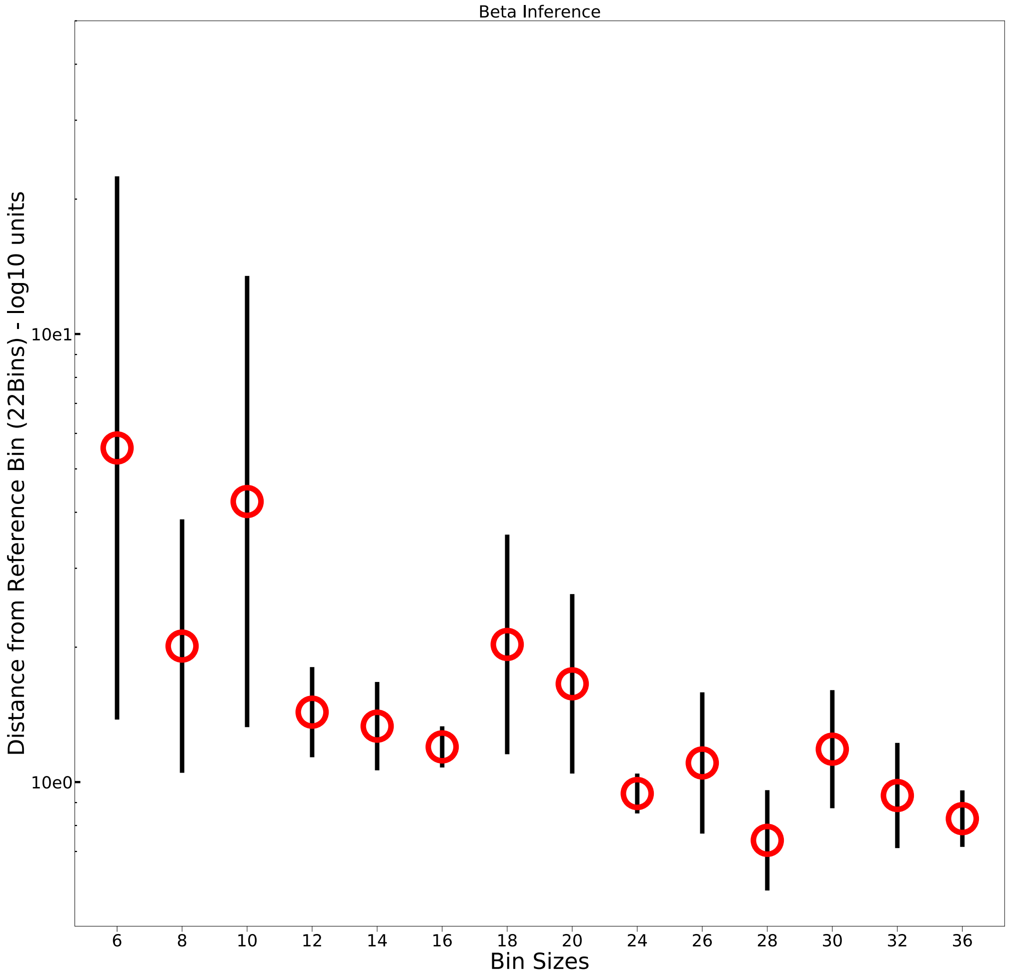


Figure S5: **Deviations in the median of the stabilization parameter $\beta$ as the number of bins changes.** The concentration manifold approach was used on the 12 CpxR regulatory positions with different number of proportional bins and the median value of $\beta$ for each bin was divided by the median of the reference bin number used in the main text. The $y$-axis represents the (log)distance in the median parameter values relative to this reference bin for bin numbers that span from 6 to 36 as detailed in the corresponding STAR Methods section. Data points represent the average of the $\log_{10}$ values for the ratios across the 12 regulatory positions with the error bars representing the standard deviation. Note that for smaller bins ($< 12$) the deviations are noticeable as the larger bin size masks the appropriate degree of curvature in the data, leading to an overestimation of $\beta$ in these cases.

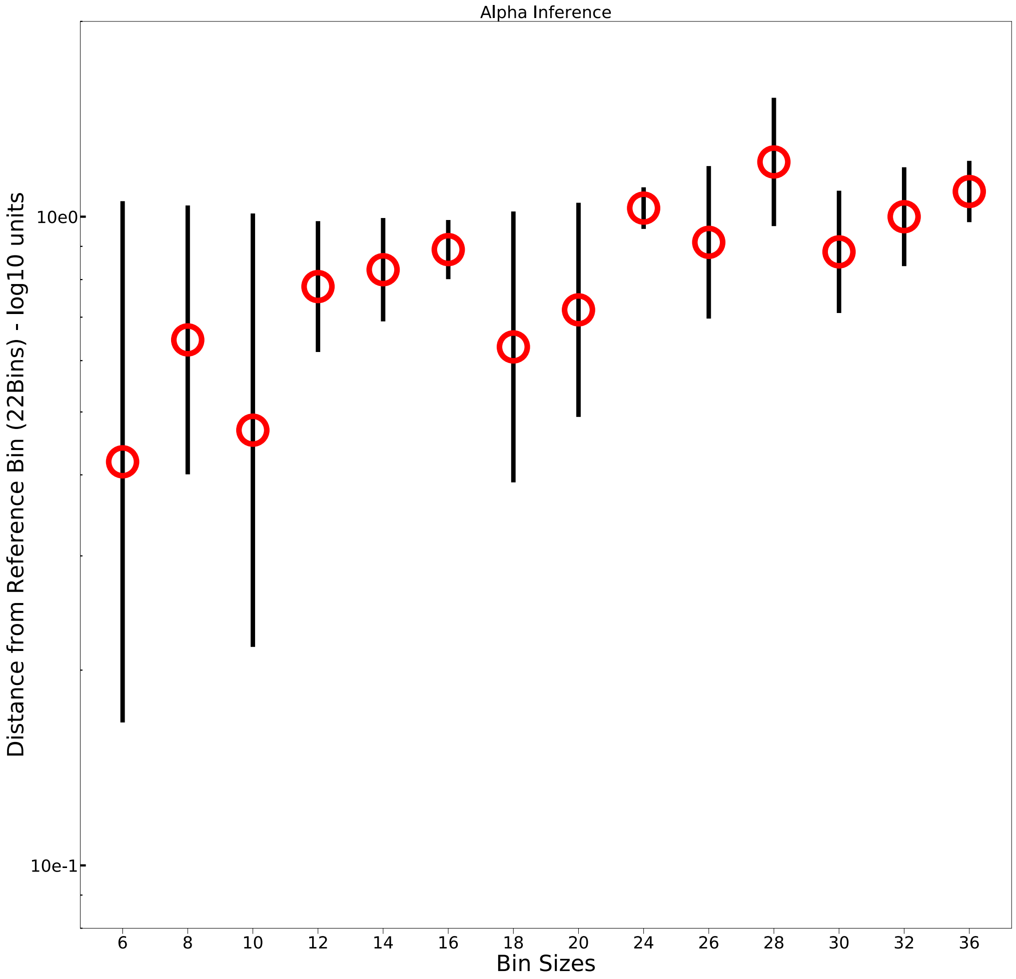


Figure S6: **Robustness of the acceleration ($\alpha$) values as the bin size is changed for concentration manifold binning.** Corresponding plot to Figure S5 for the difference in the median $\alpha$ values for different bin sizes compared to the reference bin. Data points and error bars represent the same metric as Figure S5. Note that as the values of $\beta$ in the lower bin numbers ($< 12$) were overestimated, the lower median values of $\alpha$ for these bin sizes are therefore expected. The inferred median value of $\alpha$, however, is fairly robust at larger bin numbers ($> 12$)

| TF | Primer Sequence |
|---|---|
| AcrR FP | CAT TAA AGA GGA GAA AGG TAC CAT ATG GCA CGA AAA ACC AAA CAA GAA GCG |
| AcrR RP | GCT GCT GCT TCC TTT GCT GCT GCT TCT GCT TCG TTA GTG GCA GGA TTA CGA AGC G |
| AgaR FP | CAT TAA AGA GGA GAA AGG TAC CAT ATG AGT AAT ACC GAC GCT TCA GG |
| AgaR RP | GCT TCC TTT GCT GCT GCT TCT GCC TCC CCG ACC AGA ATC ACT TCA ACC |
| ArsR FP | CAT TAA AGA GGA GAA AGG TAC CAT ATG TCA TTT CTG TTA CCC |
| ArsR RP | GCT GCT GCT TCC TTT GCT GCT GCT TCT GCA CTG CAA ATG TTC TTA CTG TCC C |
| AscG FP | CAT TAA AGA GGA GAA AGG TAC CAT ATG ATG ACG ACG ATG CTG GAA GTG G |
| AscG RP | GCT TCC TTT GCT GCT GCT TCT GCT CGC GAA GGA GCA ATG AGT G |
| BetI FP | CAT TAA AGA GGA GAA AGG TAC CAT ATG CCC AAA TTG GGG ATG C |
| BetI RP | GCT TCC TTT GCT GCT GCT TCT GCA TCG GTG GGT AGA TGC TGA G |
| CpxR FP | CAT TAA AGA GGA GAA AGG TAC CAT ATG AAT AAA ATC CTG TTA GTT G |
| CpxR RP | GCT TCC TTT GCT GCT GCT TCT GCT GAA GCA GAA ACC ATC AGA TAG C |

Table S1: **Primers for amplifying the TF gene cassettes from the MG1655 genome** FP refers to the forward primer sequence and RP refers to the reverse primer sequence. The primer pairs were designed to amplify the TF gene without the translational stop codon to allow for the TF-fusion to the mCherry gene through a linker sequence.

| Engineered TF fusion Strains | Genotype |
|---|---|
| AcrR (Constitutive) | MG1655, AcrRKO, *gspI*<>1pn25-TetR |
| AcrR (Regulation) | *gspI*<>1pn25-TetR, *ybcN*<>3*1-AcrR-AEK-mCheery, AcrRKO |
| AgaR (Constitutive) | AgaRKO, *gspI*<>1pn25-TetR |
| AgaR (Regulation) | *gspI*<>1pn25-TetR, *ybcN*<>3*1-AgaR-AEK-mCheery, AgaRPKO |
| ArsR (Constitutive) | ArsRKO, *gspI*<>1pn25-TetR |
| ArsR (Regulation) | *gspI*<>1pn25-TetR, *ybcN*<>3*1-ArsR-AEK-mCheery, ArsRKO |
| AscG (Constitutive) | AscGKO, *gspI*<>1pn25-TetR |
| AscG (Regulation) | *gspI*<>1pn25-TetR, *ybcN*<>3*1-AscG-AEK-mCheery, AscGKO |
| BetI (Constitutive) | BetIKO, *gspI*<>1pn25-TetR |
| BetI (Regulation) | *gspI*<>1pn25-TetR, *ybcN*<>3*1-BetI-AEK-mCheery, BetIKO |
| CpxR (Constitutive) | CpxRKO, *gspI*<>1pn25-TetR |
| CpxR (Regulation) | *gspI*<>1pn25-TetR, *ybcN*<>3*1-cpxR-AEK-mCheery, cpxRKO |

Table S2: **Titration strains to control the average copy number of the 6 TFs studied here.** All strains had the TF-mCherry expression cassette integrated into the *ybcN* locus.

| TF | Promoter containing sequence | Binding Sequence |
|---|---|---|
| AcrR | $P_{acrR}$ | TACATACATTCACAAATGTATGTA |
| AgaR | $P_{kbaZ}$ | CTTTCGTTTCATTTCGTTT |
| ArsR | $P_{arsR}$ | TAAGTCATATATGTTTTTGACTTA |
| AscG | $P_{ascF}$ | TGAAACCGGTTTCT |
| BetI | $P_{betI}$ | TATATTGAACGTCCAATCAA |
| CpxR | $P_{ppiA}$ | GTAAAATTAGGTAAA |

Table S3: **Binding sequences for the 6 TFs adapted from the endogenous regulated promoters in *E. coli*.**

| +1 **Binding Position** | −61 **Binding Position** | **Parent Strain** |
|---|---|---|
| *galK*<>25PDL5AcrRbs+1-YFP | *galK*<>25PDL5AcrRbs-61-YFP | *gspI*<>1pn25-TetR, *ybcN*<>3*1-AcrR-AEK-mCheery, AcrRKO |
| *galK*<>25PDL5 AgaRbs +1-YFP | *galK*<>25PDL5 AgaRbs-61-YFP | *gspI*<>1pn25-TetR, *ybcN*<>3*1-AgaR-AEK-mCheery, AgaRPKO |
| *galK*<>25PDL5 ArsRbs +1-YFP | *galK*<>25PDL5 ArsRbs-61-YFP | *gspI*<>1pn25-TetR, *ybcN*<>3*1-ArsR-AEK-mCheery, ArsRKO |
| *galK*<>25PDL5 AscGbs+1-YFP | *galK*<>25PDL5 AscGbs-61-YFP | *gspI*<>1pn25-TetR, *ybcN*<>3*1-AscG-AEK-mCheery, AscGKO |
| *galK*<>25PDL5 BetIbs+1-YFP | *galK*<>25PDL5 BetIbs-61-YFP | *gspI*<>1pn25-TetR, *ybcN*<>3*1-BetI-AEK-mCheery, BetIKO |
| *galK*<>25PDL5 CpxRbs +1-YFP | *galK*<>25PDL5 CpxRbs-61-YFP | *gspI*<>1pn25-TetR, *ybcN*<>3*1-cpxR-AEK-mCheery, cpxRKO |

Table S4: **Integrated promoter strains with Binding Sites inserted at** +1 **and** −61 **positions in the *galk* locus.** Synthetic circuits designed by cloning the binding sites for the 6 TFs listed in Table S3 at 2 distinct positions in the $P_{DL5}$ promoter sequence.

| Position Relative to TSS | Forward Primer | Reverse Primer |
|---|---|---|
| −41 | ctccgaagacctcgtgTTTACCCTTTATGCTTCCGGCT | gaattcgaagactggctcCACGAGGTGAAGCACGA |
| −43 | ctccgaagacctcgtgTGTTTACCCTTTATGCTTCCGG | gaattcgaagactggctcCGAGGTGAAGCACGAAAGG |
| −45 | ctccgaagacctcgtgCGTGTTTACCCTTTATGCTTCCG | gaattcgaagactggctcAGGTGAAGCACGAAAGGG |
| −47 | ctccgaagacctcgtgCTCGTGTTTACCCTTTATGCTTCC | gaattcgaagactggctcGTGAAGCACGAAAGGGC |
| −49 | ctccgaagacctcgtgACCTCGTGTTTACCCTTTATGC | gaattcgaagactggctcGAAGCACGAAAGGGCCTC |
| −51 | ctccgaagacctcgtgTCACCTCGTGTTTACCCTTTATGC | gaattcgaagactggctcAGCACGAAAGGGCCTCGT |
| −53 | ctccgaagacctcgtgCTTCACCTCGTGTTTACCCTTTATG | gaattcgaagactggctcCACGAAAGGGCCTCGTG |
| −55 | ctccgaagacctcgtgTGCTTCACCTCGTGTTTACC | gaattcgaagactggctcCGAAAGGGCCTCGTGATAC |
| −57 | ctccgaagacctcgtgCGTGCTTCACCTCGTGTTTAC | gaattcgaagactggctcAAAGGGCCTCGTGATACG |
| −59 | ctccgaagacctcgtgTTCGTGCTTCACCTCGTG | gaattcgaagactggctcAGGGCCTCGTGATACGC |
| −61 | ctccgaagacctcgtgCTTTCGTGCTTCACCTCGT | gaattcgaagactggctcGGCCTCGTGATACGCCTAT |
| −63 | ctccgaagacctcgtgCCCTTTCGTGCTTCACC | gaattcgaagactggctcCCTCGTGATACGCCTATTTCG |
| −65 | ctccgaagacctcgtgGGCCCTTTCGTGCTTCAC | gaattcgaagactggctcTCGTGATACGCCTATTTCGATG |
| −67 | ctccgaagacctcgtgGAGGCCCTTTCGTGCTTC | gaattcgaagactggctcGTGATACGCCTATTTCGATGGG |
| −69 | ctccgaagacctcgtgACGAGGCCCTTTCGTGC | gaattcgaagactggctcGATACGCCTATTTCGATGGGTTAATG |
| −73 | ctccgaagacctcgtgTATCACGAGGCCCTTTCGTG | gaattcgaagactggctcCGCCTATTTCGATGGGTTAATGT |
| −75 | ctccgaagacctcgtgCGTATCACGAGGCCCTTTC | gaattcgaagactggctcCCTATTTCGATGGGTTAATGTCATGG |
| −81 | ctccgaagacctcgtgAATAGGCGTATCACGAGGCCCTT | gaattcgaagactggctcTCGATGGGTTAATGTCATGGAG |
| −87 | ctccgaagacctcgtgCATCGAAATAGGCGTATCACGAG | gaattcgaagactggctcGGTTAATGTCATGGAGCTAATGGT |
| −93 | ctccgaagacctcgtgTTAACCCATCGAAATAGGCGTATC | gaattcgaagactggctcTGTCATGGAGCTAATGGTTTCTTAG |
| −99 | ctccgaagacctcgtgATGACATTAACCCATCGAAATAGGC | gaattcgaagactggctcGGAGCTAATGGTTTCTTAGACGTCG |
| −105 | ctccgaagacctcgtgAGCTCCATGACATTAACCCATC | gaattcgaagactggctcAATGGTTTCTTAGACGTCGGATG |

Table S5: **Primers used in cloning the *ccdB* cassette at the upstream and downstream locations of the $P_{DL5}$ promoter sequence.**

| *ccdB* **Cloning Strains** |
|---|
| pZS25LongUPDL5-41-ccdB-YFP |
| pZS25LongUPDL5-53-ccdB-YFP |
| pZS25LongUPDL5-55-ccdB-YFP |
| pZS25LongUPDL5-57-ccdB-YFP |
| pZS25LongUPDL5-67-ccdB-YFP |
| pZS25LongUPDL5-69-ccdB-YFP |
| pZS25LongUPDL5-73-ccdB-YFP |
| pZS25LongUPDL5-43-ccdB-YFP |
| pZS25LongUPDL5-47-ccdB-YFP |
| pZS25LongUPDL5-49-ccdB-YFP |
| pZS25LongUPDL5-51-ccdB-YFP |
| pZS25LongUPDL5-65-ccdB-YFP |
| pZS25LongUPDL5-105-ccdB-YFP |
| pZS25LongUPDL5-87-ccdB-YFP |
| pZS25LongUPDL5-93-ccdB-YFP |
| pZS25LongUPDL5-59-ccdB-YFP |
| pZS25LongUPDL5-63-ccdB-YFP |
| pZS25LongUPDL5-75-ccdB-YFP |
| pZS25LongUPDL5-81-ccdB-YFP |
| pZS25LongUPDL5-99-ccdB-YFP |
| pZS25LongUPDL5-61-ccdB-YFP |

Table S6: ***ccdB* plasmids for rapid cloning.** Plasmids used to clone the *ppiA* binding sequence for the assessing the position dependent regulatory profiles of CpxR.

| CpxR Binding Location Plasmids |
|---|
| pZS25LongUPDL5+1OCpxR-YFP |
| pZS25LongUPDL5-41OCpxR-YFP |
| pZS25LongUPDL5-43OCpxR-YFP |
| pZS25LongUPDL5-47OCpxR-YFP |
| pZS25LongUPDL5-49OCpxR-YFP |
| pZS25LongUPDL5-51OCpxR-YFP |
| pZS25LongUPDL5-53OCpxR-YFP |
| pZS25LongUPDL5-55OCpxR-YFP |
| pZS25LongUPDL5-57OCpxR-YFP |
| pZS25LongUPDL5-59OCpxR-YFP |
| pZS25LongUPDL5-61OCpxR-YFP |
| pZS25LongUPDL5-63OCpxR-YFP |
| pZS25LongUPDL5-65OCpxR-YFP |
| pZS25LongUPDL5-67OCpxR-YFP |
| pZS25LongUPDL5-69OCpxR-YFP |
| pZS25LongUPDL5-73OCpxR-YFP |
| pZS25LongUPDL5-75OCpxR-YFP |
| pZS25LongUPDL5-81OCpxR-YFP |
| pZS25LongUPDL5-87OCpxR-YFP |
| pZS25LongUPDL5-93OCpxR-YFP |
| pZS25LongUPDL5-delBS -YFP |
| pZS25LongUPDL5-99OCpxR-YFP |
| pZS25LongUPDL5-105OCpxR-YFP |

Table S7: **Cpxr binding location plasmids.** Plasmids were generated using the *ccdB* cloning vectors in Table S6. These position cloning vectors had the *ppiA* binding sequence swapped at the specified locations on the plasmid.

| Parameter | Credible Interval |
|---|---|
| $P^{\text{energy}}$ | $-5.21^{-5.076}_{-5.353}\ k_BT$ |
| $\beta^{\text{-48}}$ | $438.555^{515.780}_{339.021}$ |
| $\beta^{\text{-50}}$ | $30.305^{33.283}_{27.202}$ |
| $\beta^{\text{-54}}$ | $0.189^{0.239}_{0.139}$ |
| $\beta^{\text{-56}}$ | $50.958^{53.338}_{48.262}$ |
| $\beta^{\text{-58}}$ | $33.312^{34.958}_{31.552}$ |
| $\beta^{\text{-60}}$ | $235.952^{276.206}_{187.828}$ |
| $\beta^{\text{-64}}$ | $6421.738^{9999.904}_{5011.260}$ |
| $\beta^{\text{-70}}$ | $53.426^{56.523}_{50.045}$ |
| $\beta^{\text{-72}}$ | $13.137^{13.580}_{12.673}$ |
| $\beta^{\text{-74}}$ | $7.056^{7.314}_{6.788}$ |
| $\beta^{\text{-80}}$ | $4.464^{4.664}_{4.270}$ |
| $\beta^{\text{-82}}$ | $3.897^{4.061}_{3.737}$ |

Table S8: **Inferred parameters for the stabilization only model.** Median values of the inference chain along with the bounds that encompass the 68% Bayesian credible interval of the RNAP binding energy to the *DL5* promoter sequence along with the position specific stabilization parameters. Note that the value of the RNAP binding energy to *DL5* is roughly $1k_BT$ weaker than what was previously inferred (Brewster et al., 2012). Even with the slightly weaker binding energy, the model fails to capture the strongest activation position $-64$ along with other positions of strong curvature.

## References

Brewster, R. C., Jones, D. L. and Phillips, R. (2012). Tuning Promoter Strength through RNA Polymerase Binding Site Design in *Escherichia coli*. PLoS Computational Biology *8*, e1002811.
